# Temporal transcriptional response to latency reversing agents identifies specific factors regulating HIV-1 viral transcriptional switch

**DOI:** 10.1186/s12977-015-0211-3

**Published:** 2015-10-06

**Authors:** Narasimhan J. Venkatachari, Jennifer M. Zerbato, Siddhartha Jain, Allison E. Mancini, Ansuman Chattopadhyay, Nicolas Sluis-Cremer, Ziv Bar-Joseph, Velpandi Ayyavoo

**Affiliations:** Department of Infectious Diseases and Microbiology, Graduate School of Public Health, University of Pittsburgh/GSPH, Room A435, Crabtree Hall, 130 DeSoto Street, Pittsburgh, PA 15261 USA; Division of Infectious Diseases, Department of Medicine, University of Pittsburgh, Pittsburgh, PA 15261 USA; Molecular Biology Information Service, School of Medicine, University of Pittsburgh, Pittsburgh, PA 15261 USA; Lane Center for Computational Biology, Carnegie Mellon University, Pittsburgh, PA 15217 USA; Computer Science Department, School of Computer Science, Carnegie Mellon University, Pittsburgh, PA 15217 USA

**Keywords:** HIV-1, Latency, Transcripts, SDREM, Gene expression, Transcription factors

## Abstract

**Background:**

Latent HIV-1 reservoirs are identified as one of the major challenges to achieve HIV-1 cure. Currently available strategies are associated with wide variability in outcomes both in patients and CD4^+^ T cell models. This underlines the critical need to develop innovative strategies to predict and recognize ways that could result in better reactivation and eventual elimination of latent HIV-1 reservoirs.

**Results and discussion:**

In this study, we combined genome wide transcriptome datasets post activation with Systems Biology approach (Signaling and Dynamic Regulatory Events Miner, SDREM analyses) to reconstruct a dynamic signaling and regulatory network involved in reactivation mediated by specific activators using a latent cell line. This approach identified several critical regulators for each treatment, which were confirmed in follow-up validation studies using small molecule inhibitors. Results indicate that signaling pathways involving JNK and related factors as predicted by SDREM are essential for virus reactivation by suberoylanilide hydroxamic acid. ERK1/2 and NF-κB pathways have the foremost role in reactivation with prostratin and TNF-α, respectively. JAK-STAT pathway has a central role in HIV-1 transcription. Additional evaluation, using other latent J-Lat cell clones and primary T cell model, also confirmed that many of the cellular factors associated with latency reversing agents are similar, though minor differences are identified. JAK-STAT and NF-κB related pathways are critical for reversal of HIV-1 latency in primary resting T cells.

**Conclusion:**

These results validate our combinatorial approach to predict the regulatory cellular factors and pathways responsible for HIV-1 reactivation in latent HIV-1 harboring cell line models. JAK-STAT have a role in reversal of latency in all the HIV-1 latency models tested, including primary CD4^+^ T cells, with additional cellular pathways such as NF-κB, JNK and ERK 1/2 that may have complementary role in reversal of HIV-1 latency.

**Electronic supplementary material:**

The online version of this article (doi:10.1186/s12977-015-0211-3) contains supplementary material, which is available to authorized users.

## Background

Development of combined antiretroviral therapy (cART) has made it possible to treat and control HIV-1 infection in infected individuals. Despite these major strides in the management of HIV-1 infection, HIV-1 cure still remains a challenge. Virus persists indefinitely even when the patient is treated with cART. Studies have identified resting CD4^+^ T cells as the main reservoir for the latent virus [1–5]. Discontinuation or interruption of treatment in well-controlled subjects results in increasing virus titer and recurrence of clinical symptoms associated with HIV/AIDS. This is due to the ability of the virus to establish a state of latent infection in a small number of cells where the virus remains transcriptionally silent for long periods of time [6–8]. Currently available antiretroviral agents are effective against actively replicating virus and prevent new infection; however, they fail to eliminate the latent reservoirs. Hence there is an urgent need to develop novel therapies that can eliminate the latent viral reservoirs. It is established that CD4^+^ resting memory T cells are the latent cells in vivo [9, 10], that are non permissive for viral gene expression. However, the mechanism(s) and the factors involved in this process are not fully defined. Though resting CD4^+^ T cells are the main reservoir for the latent virus, additional reservoirs include other cell types of T cell linage, including naïve T cells; macrophages; and cells of the central nervous systems [11–15]. Various methods have been proposed to activate and kill the latently infected cells; however, these strategies have resulted in variable outcomes in clinical trials, indicating that a better understanding of the factors and/or mechanisms regulating reactivation of latent virus is needed.

Studies have shown that viral latency is facilitated in part by transcription factors such as NF-κB, AP-1, NFAT and histone modification as well as DNA methylation in HIV-1 LTR in different HIV latency cell models and in primary cells [16–21]. However, compounds/reactivators targeting histone acetylation (SAHA, suberoylanilide hydroxamic acid), NF-κB activation (prostratin) or T cell activation (α-CD3/α-CD28) do not consistently reactivate the latently infected cells in patients, suggesting that HIV-1 latency is controlled by multiple host factors and regulatory proteins. Similarly the ability of latency reversing agents to reactivate latent HIV-1 in various in vitro T cell based HIV-1 latency models are also highly variable, suggesting that multiple factors and mechanisms are involved in different cell types [22–25].

In an effort to identify the factors and their regulatory pathways involved in reactivation mediated by a specific activator, we generated and integrated genome wide transcriptome datasets using robust Systems Biology approaches. As a “proof of concept”, ACH-2 cells, a T cell line harboring a single copy of latent HIV-1 provirus, was activated with SAHA, prostratin or TNF-α and transcriptome analyses was performed at multiple time points to identify host cellular transcripts and upstream transcription factors (TFs) that regulated activation. ACH-2 cells have a mutation in TAR region affecting the Tat mediated transactivation, this helps to identify the factors involved in early stages of HIV-1 latency reversal in the absence of viral proteins including Tat, though absence of effective Tat-TAR axis can promote latency status of infected cells. Our methods were used to reconstruct dynamic signaling and regulatory networks for these activators. The reconstructed model identified several key regulators for each treatment, and the specific set of genes they control either directly or indirectly. Furthermore, specific cellular factors and signaling pathways identified by SDREM analyses are validated using small molecule inhibitors. Our combinatorial approach was able to identify the key pathways involved in latent HIV-1 reactivation. JAK-STAT appears to have a critical role in HIV-1 transcription independent of the method of reactivation. Additionally, regulatory pathways involving JNK is required for virus reactivation by SAHA in ACH-2 cells, whereas, ERK1/2 and NF-κB, pathways are involved with reactivation with prostratin and TNF-α, respectively. Evaluation using another HIV-1 latent cell line, J-Lat as well as human primary resting T cells also confirmed that many of the factors associated with latency reversing agents are similar in different cell lines, though differences are also noted. Here we have demonstrated that temporal transcriptional analysis assessed by SDREM is a useful tool to identify the key regulators and/or pathways involved in latent HIV-1 reactivation.

## Methods

### Cell culture and reagents

ACH-2, A3.01 and J-Lat cell lines were obtained through the NIH AIDS Research and Reference Reagent Program, Division of AIDS, NIAID, NIH (ACH-2 cell line from Dr. Thomas Folks [26, 27]; J-Lat Full Length Clones from Dr. Eric Verdin [28, 29]) and were maintained in RPMI containing 10 % FBS, 1 % l-glutamine and 1 % penicillin–streptomycin (GIBCO). Multiple J-Lat cell lines derived from the same parent cell line, Jurkat T cell line, were included in our study. These cell lines contain either a full length GFP reporter virus (HIV-1 ΔNΔE-GFP) (3 cell clones—FL8.4, FL9.2 and FL10.6) or LTR–Tat-IRES-GFP (5 cell clones—TG82, TGA1, TGA2, TGA7 and TGH2). SAHA (suberoylanilide hydroxamic acid), prostratin, TNF-α and PHA-M (Phytohemagglutanin-M) were obtained from Sigma-Aldrich and R&D Systems. IΚK2 inhibitor V and SB203580 were obtained from CalBiochem. FK506 (Tacrolimus), Cyclosporin A and SB600125 were purchased from Abcam Biochemicals. Rottlerin, U0126 and WP 1066 were obtained from Sigma-Aldrich, Cell Signaling Technology and Enzo Life Sciences, respectively. In time kinetic experiments, the cells were treated with a compound and cells were collected at multiple time points from the single pool to reduce variability between time points within an experiment (paired design, repeated measures).

### Purification of primary resting CD4^+^ T cells

Peripheral blood mononuclear cells were isolated from HIV-1 seronegative whole blood by Ficoll-Paque density gradient centrifugation (GE Healthcare). CD4^+^ T cells were purified by magnetic bead negative selection using a CD4^+^ T cell isolation kit (Miltenyi Biotec) and resting CD4^+^ T cells (rCD4^+^) were purified by magnetic bead negative selection using anti-CD25, anti-CD69 and anti-HLA-DR antibodies (Miltenyi Biotec).

### Primary T cell treatment, infection and stimulation

Freshly isolated rCD4^+^ T cells were cultured at a density of 1–2 × 10^6^ cells/mL in RPMI-1640 supplemented with 10 % FBS and pencillin/streptomycin/l-glutamine (100 Units/mL; 100 µg/mL; 0.292 mg/mL respectively, Life technologies) along with the chemokine CCL19 (100 nM, R&D systems), as described previously [30]. After being in culture for 2 days, rCD4^+^ T cells were infected with HIV-1_LAI_ (MOI = 1, titered on GHOST cells) for two to 3 h at 37 °C. The cells were then washed twice with media to remove any free virus and replaced back at 37 °C in fresh media. Two and four days post-infection, rIL-2 (10 U/mL, Roche) and EFV (300 nM, NIH AIDS Reagent Repository) were added to each well to inhibit any new rounds of infection that may occur. Seven days post-infection, the cells were washed again with media and plated in a 96-well plate at 100,000 cells/well. Cells were either stimulated with anti-CD3/CD28 (3 beads/cell, Life technologies) or 5 µM prostratin, and one of five inhibitors: 10 µM AZD 6244, 25 µM IΚK2-V, 50 µM SP600125, 5 µM WP1066 or 50 µM SB203580 were included at either 4 h prior to or 4 h post stimulation. Four days post-stimulation, half of the media was replaced with fresh media containing rIL-2 (10 U/mL, Roche) and EFV (300 nM).

### Flow cytometry

Surface staining of the cells was performed with CD2, CD3, CD4, CD28 antibodies or isotype controls as described [31]. For the detection of intracellular p24, fixation and permeabilization were carried out using the CytoFix–CytoPerm kit (BD Biosciences, Mountainview, CA, USA) and intracellular p24 staining was performed at room temperature for 1 h using 1 µl of anti-p24-FITC antibody (Coulter, Miami, FL, USA; clone KC47) per 10^6^ cells, followed by two washes in Perm-Wash buffer, and finally resuspended in FACS buffer. Samples were analyzed using Fortessa (BD Biosciences) with 20,000-gated events acquired for each sample, and the results were analyzed using FlowJo software (Tree Star, Inc., OR, USA).

### Real-time RT-PCR analysis

Total RNA extracted using RNeasy Mini Kit (Qiagen, CA, USA) was used to quantitate RPLPO, Multiply Spliced RNA (MS-RNA) and HIV-1 gag–pol transcripts by real-time PCR as described before [32, 33]. Briefly, a two-step RT-PCR was performed as follows: RNA was reverse transcribed using Taqman Reverse Transcription Reagents (Applied Biosystems, Foster City, CA, USA); Real-time PCR was carried out in triplicate using primer/probe sets specific for MS-RNA, HIV-1 gag-pol and ribosomal large protein (RPLPO, Applied Biosystems). The comparative C_T_ method was used to determine the relative level of MS-RNA and HIV-1 gag transcripts by normalizing to the RPLPO control transcript.

For HIV-1 RNA quantification from primary cell culture, culture supernatant samples were spun at 16,100×*g* for 70 min to pellet HIV-1 virions. HIV-1 RNA was extracted from the virions using the RNeasy PLUS Mini Kit per the manufacturers protocol (Qiagen). To quantify total HIV-1 RNA in the culture supernatant, the extracted HIV-1 RNA samples were first converted into cDNA followed by real-time PCR using the protocols previously described [34] with few modification (AffinityScript Multiple Temperature RT (Agilent technologies) was used instead of Superscript II RT). The primers and probe used to quantify HIV-1 RNA were used as described previously [35]. High copy number HIV-1 RNA transcripts were serially diluted to use as a RNA standard also as previously described [35].

### Transcriptome profiling and data analysis

Illumina HT-12 V4 array bead chips (Illumina, Inc., San Diego, CA, USA) were used for whole genome transcriptome analysis for mRNA profiling after different treatment of ACH-2 cells. Each array targets about 47,231 probes that include 28,688 well-characterized or annotated coding transcripts along with 11,121 coding transcripts with provisional annotation and remaining being non-coding transcripts and splice variants. RNA samples (1 μg) were labeled using the ‘TotalPrep RNA’ labeling kit (Ambion), reverse transcribed to cDNA; cRNA was synthesized from cDNA with labeling and hybridized onto array bead chips overnight on rocker and scanned on ‘iScan system’, according to the manufacturer’s protocols as well as standardized protocols developed by the Genomics and Proteomics Core Laboratories at the University of Pittsburgh. Datasets will be deposited in NCBI gene expression and hybridization array data repository GEO database. The data were analyzed using GenomeStudio to identify the differentially regulated gene transcripts. The data were normalized by rank invariant method and no background subtraction was included, additionally, the missing samples were excluded. For calculating differential expression, the Illumina custom model was included along with multiple testing corrections using Benjamini and Hochberg False Discovery Rate, which is a standard methodology recommended by GenomeStudio to compare paired data [36]. The differential score is a transformation of the *p* value that provides directionality to the p-value based on the difference between the average signal at time point zero versus different time points. The formula used for calculating Differential score = 10 × (Mean signal intensity at given time point (μ_t_) − Mean Signal intensity at time point 0 (μ_t0_)) × Log_10_p. A Differential score of ±13, corresponding to p < 0.05 was considered as the cut-off to identify significantly regulated transcripts.

### Gene set enrichment analysis (GSEA)

To identify the biological process/function associated at virus replication at initial virus reactivation and later productive stage, the transcriptome data was analyzed using GSEA/MSigDB (version 4.0) (http://www.broadinstitute.org/gsea/msigdb/annotate.jsp) [37, 38]. First, a list of genes (regulated by more than twofolds, with p-value <0.05) was obtained for the time point in each treatment corresponding to virus reactivation and gag production/virus release (Multiple probes for the same gene was integrated together and analyzed at gene level). The identified genes were then analyzed using GSEA, with an FDR q-value below 0.05. This represents genes coordinately regulated in predefined gene sets from various biological pathways.

### Signaling and dynamic regulatory events miner (SDREM)

To reconstruct signaling and regulatory networks activated following different treatments, we used SDREM as described [39, 40]. For the regulatory part, SDREM integrates condition specific time series gene expression data with global protein-DNA interaction data to identify bifurcation events in a time series (places where the expression of previously co-expressed set of genes diverges)–and the transcription factors (TFs) controlling these split events. While some TFs are transcriptionally activated, others are only activated post translationally via signaling networks. To identify and explain these TFs, the second part of SDREM links sources (host proteins that directly interact with the virus, in this case human proteins that were determined to physically interact with HIV-1 viral proteins [41]) to the TFs determined to regulate the regulatory network. This part of SDREM uses experimentally derived protein–protein interaction (PPI) from Biogrid and protein modification data [42] to infer such pathways—while imposing the constraint that the direction of PPI in the inferred pathways is consistent. These two parts (regulatory and signaling reconstruction) iterate a fixed number of times until the final network is obtained. See Gitter and Bar-Joseph [39] for complete details.

### Immunoblotting

ACH-2 cells or A3.01 cells were treated with SAHA, prostratin, or TNF-α, and at the indicated time point, the cells were washed twice with PBS and lysed in RIPA buffer containing 50 mM Tris (pH 7.5), 150 mM NaCl, 1 % Triton X-100, 1 mM sodium orthovanadate, 10 mM sodium fluoride, 1.0 mM phenylmethylsulfonyl fluoride, 0.05 % deoxycholate, 10 % sodium dodecyl sulfate, aprotinin (0.07 trypsin inhibitor unit/ml), and the protease inhibitors leupeptin, chymostatin, and pepstatin (1 μg/ml; Sigma). Cell lysates were clarified by centrifugation, and total cell lysates (30 μg) were separated on a 12 % sodium dodecyl sulfate–polyacrylamide gel (SDS-PAGE) electrophoresis gel, transferred, and immunoblotted with anti-NT5C3 (santa cruz biotechnology) or anti- tubulin antibodies. The blots were developed using an ECL kit (Amersham Biosciences, Piscataway, NJ, USA).

### Gag p24 ELISA

Following different treatment of ACH-2 and J-Lat cells, supernatants were collected and analyzed for the amount of HIV-1 p24 Gag. Gag (p24), was measured by using enzyme-linked immunosorbent assay (ELISA) kit (Zeptometrix, Buffalo, NY, USA) according to the manufacturer’s protocol.

### Statistical analysis

The results from p24 ELISA, comparative reactivation studies, and inhibitor experiments are expressed as mean ± standard deviation. The data are analyzed using the Student’s t test for paired samples.

## Results

### Multiple complementary factors are involved in reversal of latent HIV-1

**Fig. 1:**
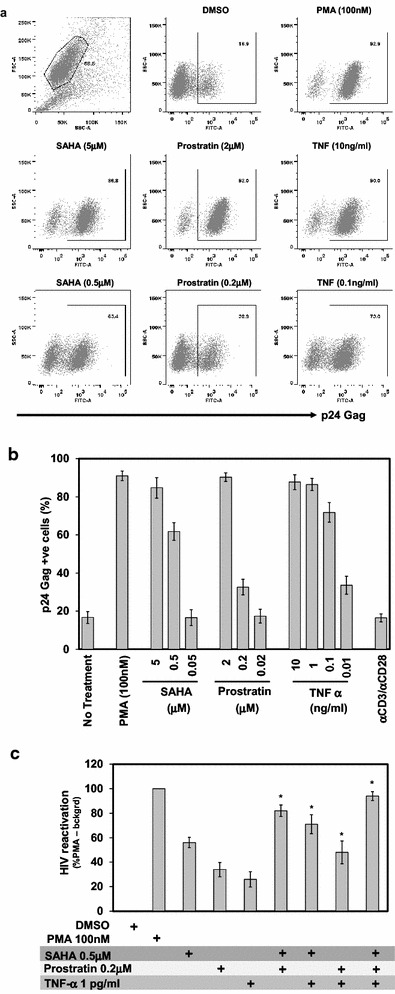
Dose dependent reversal of latent HIV-1 in ACH-2 cells by SAHA, prostratin and TNF-α. **a** ACH-2 cells were treated with different reactivating agents for 18 h and p24-Gag was estimated by intracellular staining. A representative figure denoting intracellular HIV-1 p24 Gag detected by flow cytometry (N = 4) is shown here. Viable cells were gated based on side and forward scatter *dot plot* and the p24 positive cells were detected by anti-p24-FITC antibody **b** The percentage of HIV-1 Gag-p24 positive cells from multiple experiments (N = 4) are shown, to demonstrate the dose dependent response of ACH-2 to different activating reagents. *Error bars* represent standard deviation. **c** Effect of combination of reactivating agents. For comparison of results across samples from multiple experiments, HIV reactivation observed upon PMA (100 nM) treatment minus background (DMSO) was considered as 100 %. *Error bars* represent standard deviation (N = 4). *p < 0.05 by student t test

Comparative studies using multiple activators to reactivate HIV-1 from latency have resulted in different levels of virus reactivation [43–46], suggesting a role for multiple factors in virus reactivation. Here we used a well-characterized ACH-2 T cell line based HIV-1 latency model as our model cell line to validate the Systems Biology approach to identify the cellular factors and pathways involved in reversal of latent HIV-1 by SAHA, prostratin and TNF-α. ACH-2 cells were treated with different concentrations of SAHA, prostratin, or TNF-α for 18 h. Alternatively, the cells were also activated using αCD3/αCD28 antibody for 3 days, and virus reactivation was measured by intracellular p24 staining (Fig. 1a). Phorbol 12-myristate 13-acetate (PMA) was used as a positive control. In the presence of vehicle (DMSO) control alone, 13–20 % of cells are positive for p24 Gag as measured by flow cytometry. PMA (positive control), which is a potent activator of protein kinase C (PKC) reactivated 90–95 % of the cells in 18 h, suggesting that the ACH-2 cells have a wide dynamic range (Fig. 1a) and can serve as a sensitive model for estimating the potency of HIV-1 latency reactivators and to elucidate the contribution of cellular factors and signaling pathways in HIV-1 transcription regulation. Addition of clinically relevant concentration of SAHA (0.5 μM) reactivated 57–65 % of ACH-2 cells as detected by positive p24 Gag staining, and increasing concentrations (5 µM) of SAHA resulted in ~95 % of them positive for p24 Gag (Fig. 1b). Similarly we observed a dose dependent increase in the reactivation of latent HIV-1 in ACH-2 cells, with increasing concentrations of prostratin and TNF-α as well. At 0.2 μM concentration, prostratin activated 32–37 %, whereas, at 2 μM, the percentage positive cells increased to 87–94 %. TNF-α, a potent activator of latently infected cells exhibited similar activation levels at 10 ng/ml concentration. Interestingly, stimulation with and anti-CD3 and anti-CD28 antibodies failed to activate significant amount of the latent virus in ACH-2 cells (Fig. 1b). Flow cytometry analyses of surface expression of CD3, CD28 and CD2 suggested that only less than 15 % of ACH-2 cells expressed CD3 and CD28 molecules, and less than 10 % of the cells express CD2 (Additional file 1: Fig. S1) that are required for TCR mediated activation, respectively. These results correlate with the absence/minimal response observed in ACH-2 cells when treated with αCD3/αCD28 antibodies.

To assess whether these activators could have an additive effect in reversal of latent virus, ACH-2 cells were treated with a combination of submaximal concentrations of these compounds to reactivate latent virus (Fig. 1c). Results indicate that combining SAHA and prostratin resulted in an increase in percentage of cells expressing p24 Gag. Treatment with SAHA or prostratin alone, showed 57–65 and 32–37 % of p24 positive cells, respectively, whereas a combination of these two activators resulted in 87–92 % of p24 positive cells. These results suggest an additive effect, implying that independent cellular factors are involved in SAHA and prostratin mediated latent HIV-1 reactivation and that these factors could complement each other to potentiate the effect. A similar increase in percentage of p24 Gag positive cells was observed when SAHA was combined with TNF-α, an increase from 23–28 to 83–87 % p24 positive cells. Similarly, when prostratin and TNF-α were combined together, the p24 positive cells increased to 46-62 %, a lesser extent than the additive effect, suggesting that prostratin and TNF-α may share few common cellular factors and pathways in virus reactivation. Combining SAHA, prostratin and TNF-α together resulted in maximum reactivation (93–97 % p24 positive cells), similar to that observed in PMA treated positive control. Collectively, these results suggest that HIV-1 reactivation from latency is mediated by multiple pathways involving different cellular factors that are complementary as well as common depending on the context of the activator used.

### Kinetics of latent HIV-1 reactivation in ACH-2 cells indicates that transcriptional reactivation of viral RNA is an early event

**Fig. 2:**
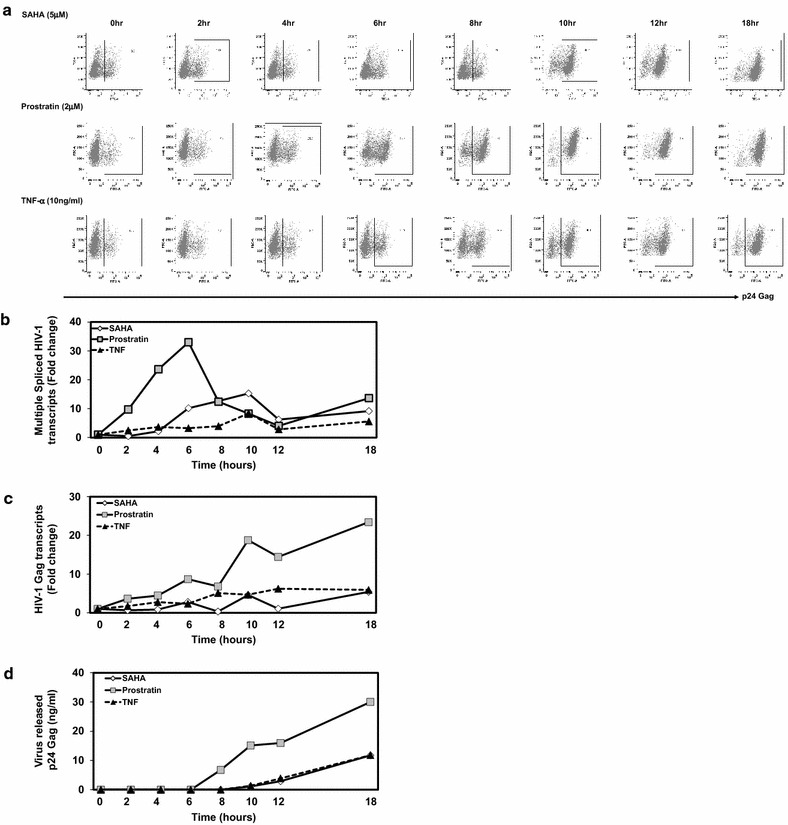
Time kinetics of p24 gag protein expression, synthesis of viral transcripts and virus release. **a** HIV-1 Gag positive cells were detected by intracellular staining for p24-Gag protein at indicated time points following treatment of ACH-2 cells with SAHA, prostratin or TNF-α. **b** Multiply spliced HIV-1 RNA transcripts, and **c** Unspliced HIV-1 (Gag-pol) RNA transcripts were detected by Real time PCR at indicated time points. The average fold changes in RNA transcripts over time zero observed in two independent experiments done in triplicates is shown. RPLPO (large ribosomal protein) was included for normalization. **d** The amount of virus released in the supernatant was detected by p24 ELISA and the p24 value at time zero was subtracted from individual time points to calculate the new virus released over time. The average of two independent experiments done in triplicate is shown

The kinetics of latent viral reactivation were assessed to understand the timing and sequence of events leading to the transcriptional switch from latency to productive infection and virus release. ACH-2 cells were treated with SAHA, prostratin or TNF-α and synthesis of viral transcripts and virus production/release was measured at 2, 4, 6, 8, 10, 12 and 18 h post activation (Fig. 2b–d). Gag (p24) positive cells were assessed by p24 intracellular staining and flow cytometry (Fig. 2a). Intracellular staining for p24 Gag indicates that ACH-2 cells treated with SAHA resulted in a shift from background level of ~20 to ~80 % 10 h post treatment and reached the maximum of 95 % with associated increase in MFI, suggesting that SAHA induced viral Gag production required 10 h. In case of prostratin, a less dramatic shift in cells expressing p24 Gag was seen in 6–8 h, whereas, TNF-α exhibited a slow increase at 6 h post stimulation and reached the maximum at 12 h. Together, these results support that SAHA, prostratin and TNF-α might utilize diverse mechanisms, with varying kinetics leading to reactivation of HIV-1 in ACH-2 cells.

To understand the time kinetics required for HIV-1 transcriptional reactivation in these cells, multiply spliced viral transcripts and unspliced gag RNA was assessed over time (Fig. 2b). Total RNA was extracted from part of the cells collected at the same time points to assess MS-RNA and unspliced Gag RNA (Fig. 2b, c) and virus released in the supernatant was measured by p24 ELISA (Fig. 2d), as described in materials and methods. Results indicate that prostratin initiated virus transcription within 2–4 h and that the peak expression of MS-RNA was reached at 6 h. Six hours post treatment there was a gradual decrease in the multiply spliced RNA and a corresponding increase in full-length gag transcripts occurred (Fig. 2b, c). The lesser fold change in cellular gag transcripts can be attributed to the packaging of full length gag transcripts in virus particles and their release in the supernatant, as measured by an increase in p24 virus Gag (Fig. 2d), resulting in sinusoidal curves of gag transcripts. In case of SAHA, we observed an increase in multiply spliced viral transcripts around 4–6 h, which was to a lesser extent than the fold change observed in prostratin. In case of gag transcripts, the increase was also to a lesser extent in comparison to prostratin occurring around 10 h. This correlates with the detection of p24 Gag by flow cytometry. The release of virus particles in SAHA as measured by an increase in p24 Gag protein in the supernatant was observed around 10 h. TNF-α resulted in an increase in multiply spliced transcripts by 6 h though the increase was very modest in multiple experiments; similarly, the increase in gag transcripts was observed around 10 h. Virus released in supernatant in TNF-α treatment of ACH-2 cells was similar to SAHA, where an increase in p24 was detected by 10 h and continued to increase over time. These results suggest that HIV-1 reactivation at transcription level in ACH-2 cells is an early event occurring within the initial 4–6 h post reactivation. Additionally, we noted that reactivation of latent virus in ACH-2 cells is a sequential process similar to acute HIV-1 infection in T cells, where the initial transcription initiation is followed by transcription of multiply spliced viral RNA transcripts, and a subsequent shift to full length gag transcripts and the final stage of viral RNA packaging and virus release.

### Transcriptome analysis to identify specific factors involved in virus reactivation

**Fig. 3:**
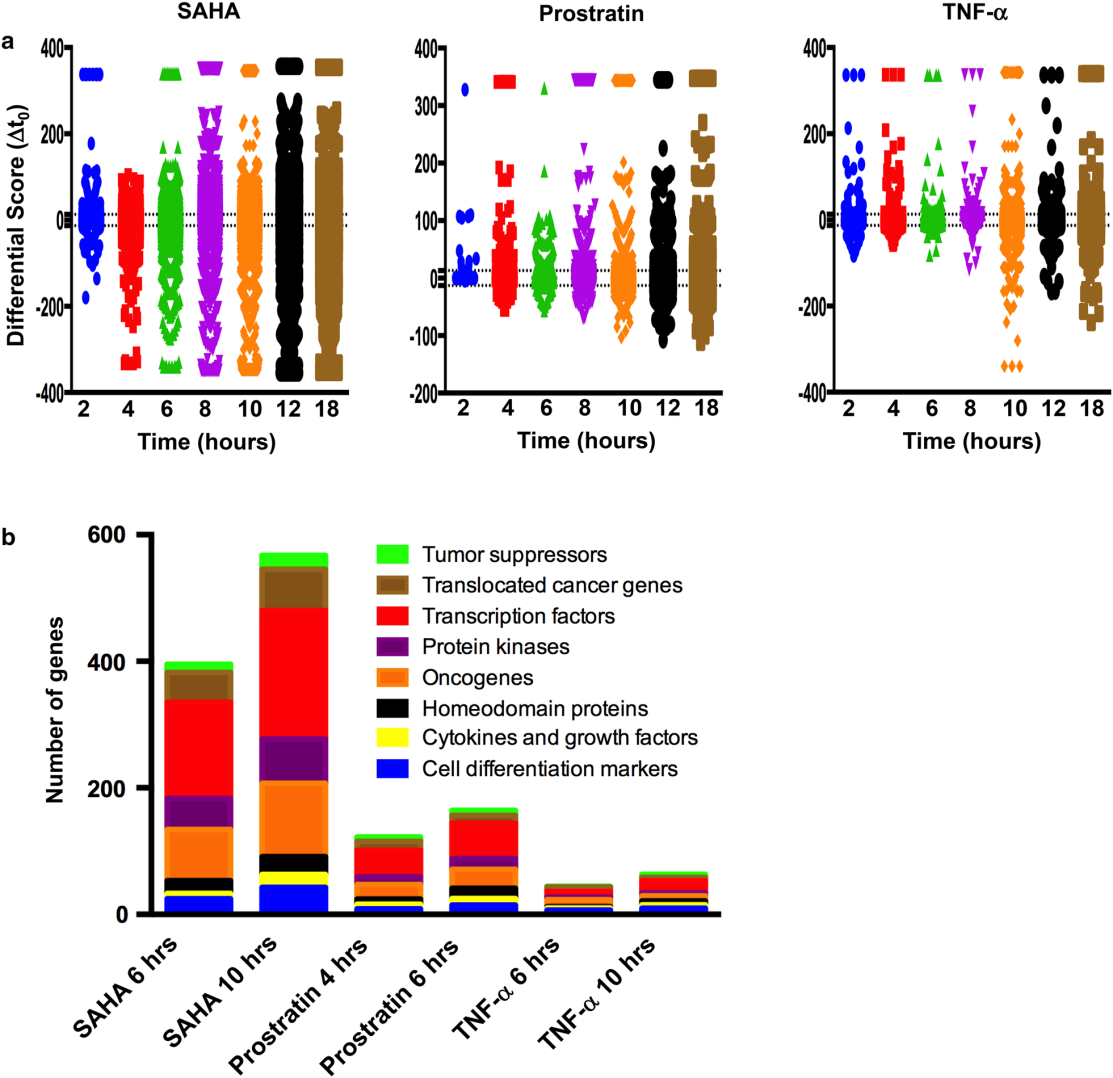
Analysis of whole genome transcriptome data. **a** Time kinetics of whole genome transcriptome analysis in ACH-2 cells treated with SAHA, prostratin and TNF- α. Genome Studio was used to analyze transcriptome data obtained from Illumina HT-12 V4 array bead chips for ACH-2 cells treated with different reactivating agents at indicated time points. A differential score of ±13 (*dotted line*) corresponding to a p < 0.05 was used to identify the significant genes. Each data point corresponds to a differential score of an individual transcript calculated from results obtained in two independent experiments. **b** Dysregulated genes (p-value <0.05 and fold change ±2) from GenomeStudio transcriptome analysis were analyzed using MSigDB version 4.0 (http://www.broadinstitute.org/gsea/msigdb/annotate.jsp). The stacked *bar graph* represents the number of genes in each functional category for different time points in ACH-2 cells treated with SAHA, prostratin or TNF-α

In an effort to identify the cellular factors and signaling pathways that are modulated following treatment of ACH-2 cells with different reactivating agents, transcriptome analyses were performed at multiple time points following treatment of cells with reactivating agents. Results indicate that multiple cellular transcripts were altered over time (Fig. 3a; Table 1). Interestingly, significant changes in cellular transcripts were observed within 2 h post treatment, with the largest changes induced by SAHA—264 host cellular gene transcripts were identified as significantly upregulated and 88 transcripts significantly downregulated. Prostratin, significantly increased the transcripts for 14 genes while none of the transcripts were significantly decreased by 2 h. For TNF-α, 83 gene transcripts were significantly upregulated and 46 cellular transcripts were significantly downregulated at this time point. Evaluation of cellular transcriptome data over time indicates that, the changes in cellular transcripts are the greatest with SAHA and relatively lesser with prostratin and least with TNF-α. Though the number of transcripts that were altered in either direction (up/down regulated) were comparable across all treatments, for SAHA the differential score for transcripts (a measure of statistical significance of the change and fold change in transcript levels) was symmetrically distributed in both the positive and negative direction, whereas, with prostratin and TNF-α, there were more cellular transcripts that were significantly upregulated than those that were down regulated.

**Table 1:** Differentially regulated cellular transcripts in ACH-2 at indicated time points following treatment with SAHA, prostratin or TNF-α

	2 h	4 h	6 h	8 h	10 h	12 h	18 h
SAHA	264	627	1347	2401	1650	2729	2511
	*88*	*400*	*738*	*1092*	*1450*	*1589*	*1852*
Prostratin	14	105	119	238	348	379	648
	*0*	*22*	*33*	*107*	*361*	*436*	*626*
TNF-α	83	68	54	66	316	119	243
	*46*	*34*	*50*	*41*	*458*	*116*	*308*

Comparison of differentially regulated cellular transcripts at multiple time points in comparison to time 0 (before addition of latency reversing agents) in ACH-2 cells. A differential score of ±13 corresponding to a p < 0.05 was used to identify the significant genes. The number of cellular gene transcripts that are significantly upregulated are in roman and that which are significantly downregulated are italicized

A single copy of HIV-1 in ACH-2 is integrated in the intron between exons 5 and 6 in NT5C3A, variant 1 on chromosome 7 [47]. Analysis of transcriptional changes in cellular genes around this region clearly identifies that NT5C3 as the only host gene that is consistently induced with the reactivation of the virus in ACH-2 cells to 7.4- 18.5 fold. None of the closely associated gene transcripts namely RP9, BBS9, FKBP9 or RP9P changes more than 1.4 to 0.9 fold with reversal of HIV-1 latency (Additional file 2: Table S1, Additional file 3: Fig. S2A). Time kinetics of changes in NT5C3 transcripts identifies that transcripts are induced as early as 2 h following treatment of ACH-2 cells with prostratin and TNF-α, and the levels continue to increase over time. With SAHA an increase in NT5C3 transcript is noticed at 4 h and this increase remains progressive and sustained, finally resulting in highest fold change of 18.5 in comparison to prostratin (12.6 fold change) and TNF-α (7.4 fold change) (Additional file 3: Fig. S2B). Induction of NT5C3 transcripts as detected by RNA hybridization in Illumina HT-12 chip was also detected at the protein level by western blot using NT5C3 specific antibody (Additional file 3: Fig. S2C). It is observed that the NT5C3 protein is not present in the media control, whereas detected upon treatment with prostratin, SAHA or TNF-α. Comparison of changes in protein level over time with the parent cell line, A3.01 cells, indicates that NT5C3 is expressed in A3.01 cells even in the absence of latency reactivating agents, Additional file 3: Fig. S2C, lane 5, suggests that, the integration of HIV-1 virus and its latent state in ACH-2 cells is associated with suppression of host cellular gene, NT5C3. With the reactivation of latent HIV-1 with either prostratin, SAHA or TNF-α is associated with reversal of this inhibition of NT5C3 expression.

Gene expression data sets were assessed to identify the pathways or major functions that are targeted by the reactivators using GSEA as described [37]. Results presented in Fig. 3b focused on time points corresponding to the expression of viral transcripts and Gag synthesis/release indicate that the differentially expressed genes prior and/or during the time point represent primarily genes belong to transcription factors, protein kinases, cell differentiation markers and cancer related genes as well as cytokines and growth factors.

### Transcriptional regulators and pathways involved in the reactivation of HIV-1

**Fig. 4:**
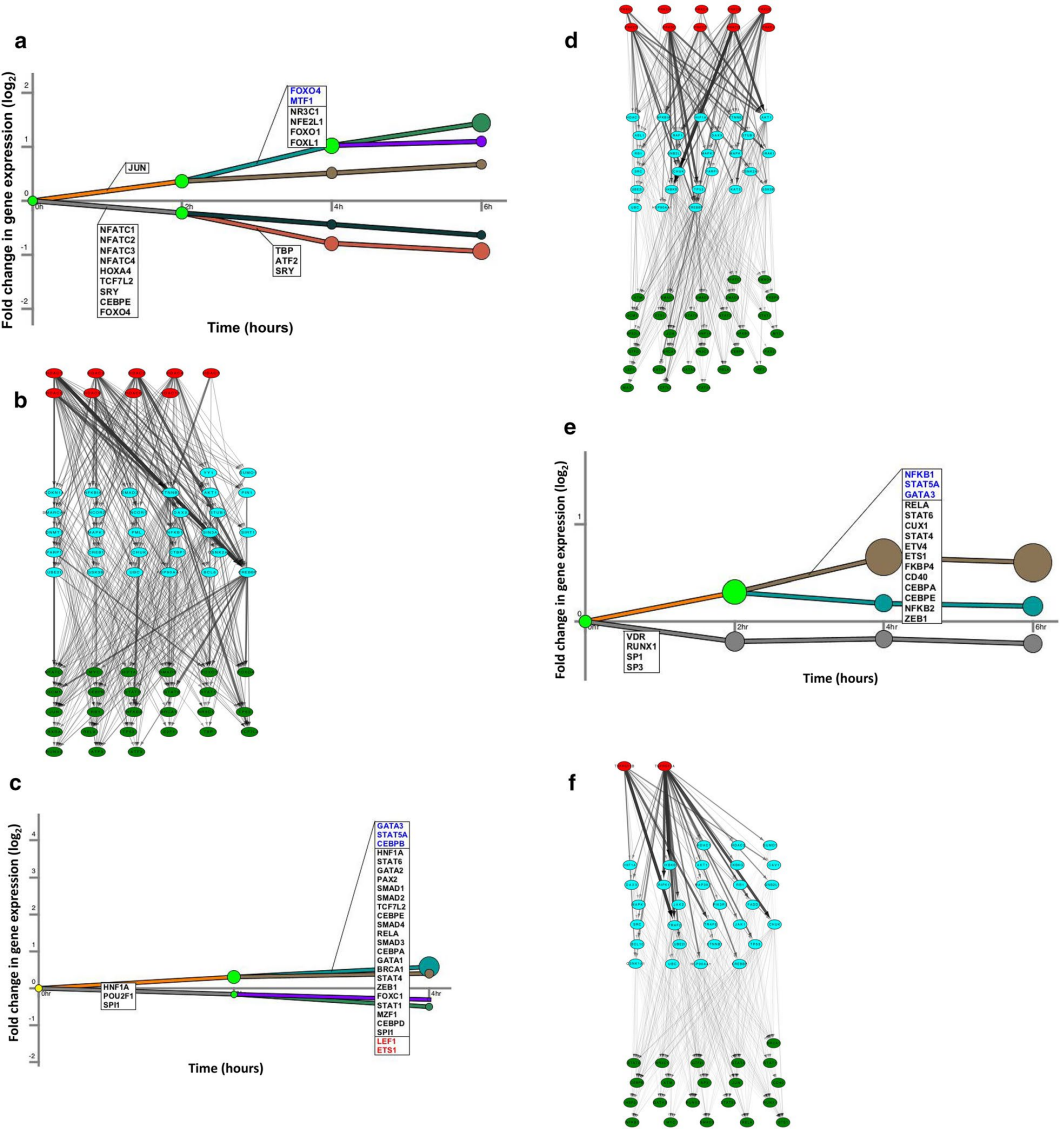
Reversal of latent HIV-1 model—SDREM analysis. Whole genome transcriptome data in ACH-2 cells at multiple time points were analyzed by SDREM for **a**, **b** SAHA; **c**, **d** Prostratin; **e**, **f** TNF-α. **a**, **c**, **d** represent the regulatory part of the model, where each path represents a collection of gene expression profiles; x-axis denotes the time points for each treatment when the gene expression was measured and the y-axis shows log_2_ fold change in expression. TFs that are predicted to control the split are included at indicated time points (TFs are included only the first time they are active along a regulatory path). TFs in* red* or* blue* indicate that transcripts of these TFs were also observed to be significantly increased or decreased respectively. The size of the node indicates the relative number of genes regulated. **b**, **d**, **f** represent oriented interaction network starting from upstream proteins (source; *red*), predicted signaling proteins (*blue*) and active TFs (*green*). The* boldness* of the edge between two nodes, indicate the number of pathways between the two

Next, to identify the upstream regulators including the transcription factors and their associated signaling molecules, the time series transcriptome data corresponding to each of these treatments were used to reconstruct dynamic signaling and regulatory networks using SDREM (Fig. 4a–f). Based on our observations from viral transcriptome analysis, the reactivation of latent HIV-1 in ACH-2 cells was initiated as early as 4–6 h following treatment with SAHA, prostratin or TNF-α. Hence for SDREM analysis, the transcriptome data were divided into two sets—(1) initial reactivation phase, corresponding to time points before the expression of viral transcripts; and (2) virus production phase, associated with time points post viral transcripts synthesis. The transcriptome changes in the initial reactivation phase are key for switching on the latent HIV-1 transcription and are the consequence of reactivating agents, while changes in the virus production phase are due to the combined effects of reactivating agents and viral products. Results from the SDREM analyses corresponding to initial reactivation phase (6 h post treatment) with SAHA suggest that TFs–JUN, NFATC1-4, HOXA4, TCF7L2, SRY, CEBPE, and FOXO4 are responsible for changes observed in the cellular transcripts in the initial 2 h. Similarly FOXO4, MTF1, NR3C1, NFE2L1, FOXO1, FOXL1, TBP, ATF2 and SRY were predicted to be responsible for changes observed in the cellular transcripts observed between two and 4 h. No additional changes in TFs were observed at 4–6 h. Identification of regulatory networks using the transcriptome data from ACH-2 cells activated with SAHA is presented in Fig. 4a. As SAHA is well characterized as an inhibitor of histone deacetylase enzyme (HDAC), we included all isoforms of HDACs as the source molecules for the SDREM analysis. The downstream nodes representing the signaling and regulatory components constructed by SDREM are represented in Fig. 4b; Table 2. This includes the top 30 factors that are predicted to be involved in signal transduction mediated downstream events of HDACs following treatment of ACH-2 cells with SAHA.

**Table 2:** List of top 30 cellular factors involved in SAHA, prostratin or TNF-α induced latent HIV-1 reactivation in ACH-2 cells

SAHA	Prostratin	TNF-α
UBC	UBC	UBC
CREBBP	TP53	TP53
JUN	AKT1	TRAF2
TP53	CREBBP	BRCA1
RB1	BRCA1	JUN
RELA	JUN	HSP90AA1
BRCA1	NR3C1	RELA
NR3C1	SMAD3	CEBPB
CTNNB1	RELA	HDAC1
SMAD3	MYC	SMAD3
CEBPB	HDAC1	IKBKB
MYC	IKBKB	STAT3
TBP	CTNNB1	CREBBP
ATF2	STAT3	NR3C1
STAT3	CHUK	CHUK
ATF3	HSP90AA1	ETS1
HIF1A	CEBPB	NFKB1
TP63	SRC	MYC
FOXO1	SMAD2	STAT1
RXRA	ETS1	RUNX2
STAT2	SMAD4	SUMO1
FOXO4	NFKB1	STAT5A
NFATC1	STAT1	STAT6
RUNX2	GSK3B	GTF2I
E2F1	MAPK1	SP3
STAT1	STAT5A	ATM
AKT1	MAX	TRAF6
NFKB2	ATM	RUNX1
TCF7L2	RB1	UBE2I
DAXX	LEF1	GATA3

Proteins are ranked based on the “path flow” going through a protein. The path flow $f$ through a protein $n$ is defined as follows—$f\left( n \right) = \mathop \sum \nolimits_{p \in P} I\left( p \right) \cdot h_{p}$ where $P$ is the set of signaling pathways predicted by SDREM that contain the protein $n$ and $h_{p}$ is the confidence in the existence of path $p$ which is a product of the confidence in each individual edge of the path (The confidence is a number between 0 and 1). The proteins are ranked in descending order of the path flow going through them

Similar analyses were performed for prostratin induced reactivation in ACH-2 cells (Fig. 4c, d). Results indicate that TFs—HNF1A, POU2F1, SPI1, ETS1, LEF1, POU2F1, CEBP, SMAD, TCF7L2, RELA, GATA, STATs, BRCA1, ZEB1, FOXC1 and MZF1 are regulating the transcriptional changes observed at 4 h post prostratin treatment (Fig. 4c). It is also noted that the expression levels of ETS1 and LEF1 are increased and the expression of GATA3, STAT5A and CEBPB is reduced at this time point. As prostratin is an activator of Protein Kinase C (PKC), PKC was included as a source and the regulatory pathways and factors that were predicted to mediate the changes observed in the regulatory models are identified in Fig. 4d. The top 30 factors predicted by SDREM as critical for prostratin effects in ACH-2 cells are also presented in Table 2. Results indicate that MAPK1, SMAD2/4, SRC, MAX, GSK3B, and LEF1 are identified as unique factors involved in prostratin induced signaling and predict PRKCQ as the main driving factor responsible for the changes observed in cellular transcripts during early time duration of 0–4 h following prostratin treatment.

Unlike prostratin, TNF-α exhibited delayed reactivation kinetics in ACH-2 cells. SDREM analysis of time kinetic data obtained from TNF-α treated cells presented in Fig. 4e, suggests that TFs- VDR, RUNX1, SP1 and SP3 are contributing to the differential regulation of cellular transcripts observed at 2 h. TFs related to NF-κB—NF-κB1, NF-κB2, RELA; JAK-STAT related factors—STAT4, STAT5A, STAT6; CEBPA, CEBPE, GATA3, CUX1, ETV4, ETS1, FKBP4, CD40, ZEB1 are identified to be responsible for changes observed in the cellular transcripts at 2 and 4 h. The regulatory pathways and factors likely responsible for changes in TFs originating from TNFRSF1A and TNFRSF1B are indicated in Fig. 4f and Table 2. TFs, Jun, Myc, RelA, STAT1/3, TP53, NR3C1, CEBPB, and CREBBP, which are known to bind to HIV-1 LTR were present in all three treatments. Together these results suggest that specific transcription factors and their associated cellular signaling pathways are involved in HIV-1 reactivation in the ACH-2 cell line. It should be noted that SDREM identifies the upstream regulatory factors as the most probable factors responsible for changes observed in the transcripts at the identified time point and hence changes (activation/inhibition) in these upstream factors occur prior to the time points when the changes in transcripts were evaluated.

### Validating the predicted cellular factors and TFs in latent HIV-1 reactivation in ACH-2 cells by specific inhibitors

**Fig. 5:**
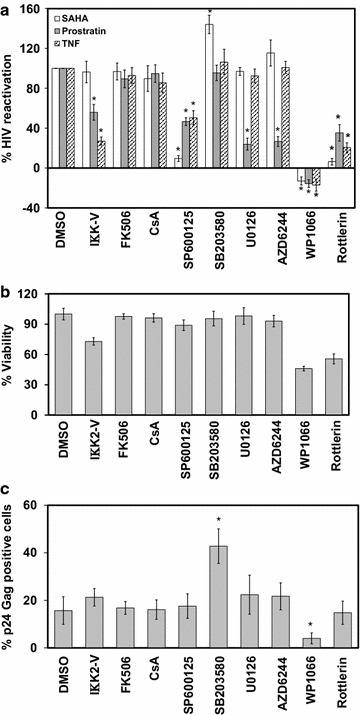
Specific cellular signaling pathways with associated host cellular factors are involved in reactivation of latent HIV-1 in ACH-2 cells. **a** ACH-2 cells were pretreated with specific inhibitors IΚK2 inhibitor V (50 µM), FK506 (10 µM), Cyclosporin A (10 µM), SP600125 (50 µM), SB203580 (50 µM), U0126 (50 µM), AZD6244 (10 µM), WP1066 (5 µM), Rottlerin (50 µM), or vehicle control (DMSO), and 4 h later activated with SAHA (1 µM, *white bars*), prostratin (1 µM, *grey bars*) or TNF-α (0.1 ng/ml, *bars* with diagonal *line* *upwards*). HIV reactivation was estimated at 12 h following reactivation, by intracellular p24 Gag staining by flow cytometry. For comparison of results across samples from multiple experiments, HIV-1 reactivation observed in vehicle control pretreatment was considered as 100 % and the background (no reactivating agent) as 0 %. *Error bars* represent standard deviation (N = 3), *p < 0.05. **b** At the end of 16 h following addition of inhibitors, the live cells were evaluated by Trypan blue staining. The percentage of viable cells was calculated by subtracting the dead cells from total cells divided by total cell count. **c** The amount of p24-Gag positive cells was estimated by intracellular p24-Gag staining and flow cytometry. *Error bars* represent standard deviation (N = 3), *p < 0.05

SDREM analysis has identified both common and unique TFs as the key regulators of cellular transcripts in ACH-2 cells, when treated with SAHA, prostratin and TNF-α. Based on these predictions we performed follow up experiments to validate the role of predicted factors in reactivation of latent HIV-1 in ACH-2 cells. First, we evaluated the ability of IΚK2 inhibitor V (NF-κB inhibitor); Tacrolimus (FK506), Cyclosporin A (CsA)—NFAT inhibitors; SP600125 (JNK inhibitor); SB203580 (p38 inhibitor); U0126 and AZD6244—ERK1/2 inhibitor; WP1066 (JAK-STAT inhibitor); and Rottlerin (PKC inhibitor) to inhibit SAHA, prostratin and TNF-α mediated reactivation of HIV-1. ACH-2 cells were pretreated with inhibitors for 4 h and activated with SAHA, prostratin or TNF-α and the p24 positive cells were assessed by flow cytometry (Fig. 5a). It is observed that both Rottlerin and SP600125 completely abrogate SAHA induced virus reactivation suggesting that the PKC→JNK→JUN/ATF pathway with related downstream TFs, has a major role in SAHA induced HIV-1 reactivation in these cells, as predicted by our model (Fig. 4a, b; Table 1). Interestingly, p38 inhibitor, SB203580 further increased the percentage of reactivated HIV-1 virus (Fig. 5a), suggesting that SB203580 reactivates latent HIV-1 using cellular pathways much different from those used by SAHA.

In the case of prostratin, IKK2 inhibitor V, SP600125, U0126, AZD6244 and Rottlerin have a significant effect in preventing virus reactivation, suggesting that prostratin uses multiple alternate pathways involving NF-κB, JNK, ERK1/2 and PKC to induce HIV-1 reactivation (Fig. 4c, d). These results correlate with the SDREM prediction for prostratin suggesting a major role for ERK1/2. Similarly, SDREM analysis was accurate in identifying factors involved in TNF-α induced HIV-1 reactivation, which involves NF-κB, JNK and PKC, but not NFAT. p38 inhibitor did not increase prostratin or TNF-α mediated HIV-1 reactivation. The percentage of cells expressing p24 was significantly below the background expression observed with DMSO, suggesting that the JAK → STAT pathway is involved in regulating HIV-1 LTR activity. Irrespective of reactivation agents, STATs were also identified as a common factor in SDREM analysis for all these activating agents. Furthermore, it is important to note that no cellular toxicity was observed in these cells with these inhibitors, with the exception of WP1066 and Rottlerin, which showed 40–60 % reduction in cell viability (Fig. 5b); therefore only live cells were gated in flow cytometry based assessment of p24 positive cells. Additionally, dose dependent inhibitory effects can be observed with WP1066 and Rottlerin, and consistent inhibition was noted at lower concentration, when the associated cellular toxicity was minimal (Additional file 4: Fig. S3).

ACH-2 cells were incubated with the inhibitors in the absence of reactivating agents, to evaluate whether these inhibitors could potentially modulate HIV-1 transcription. Results indicate that only the p38 inhibitor–SB203580 and JAK-STAT inhibitor–WP1066 significantly altered the HIV-1 transcription (Fig. 5c). WP1066 reduces the basal level HIV-1 transcription in ACH-2 cells suggesting a role for STAT in HIV-1 transcription. SB203580 increased HIV-1 transcription from 15 to 42 % at 16 h post treatment, suggesting that p38 inhibitor SB203580 could directly reactivate latent HIV-1 in ACH-2 cells.

### Validation of signaling pathways and regulatory networks in J-Lat latent cells

**Fig. 6:**
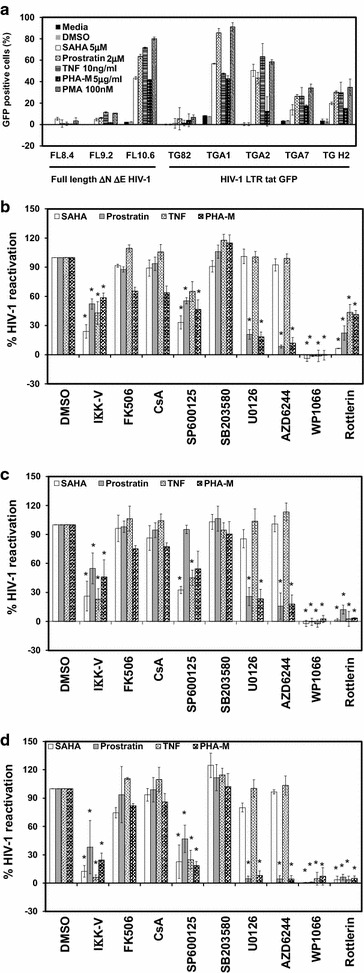
Host signaling pathways and regulatory cellular factors involved in reactivation of HIV-1 transcription in J-Lat cells. **a** Eight different clones of J-Lat cells were treated with different reactivating agents or DMSO for 18 h and the percentage of GFP positive cells was estimated by flow cytometry. *Error bars* represent standard deviation (N = 3). Cell lines **b** J-Lat FL10.6, **c** J-Lat TGA1, and **d** J-Lat TGA2 cells were pretreated with specific inhibitors IΚK2 inhibitor V (50 µM), FK506 (10 µM), Cyclosporin A (10 µM), SP600125 (50 µM), SB203580 (50 µM), U0126 (50 µM), AZD6244 (10 µM), WP1066 (5 µM), Rottlerin (50 µM), or vehicle control (DMSO), and 4 h later activated with SAHA (1 µM, *white bars*), prostratin (1 µM, *grey bars*) or TNF-α (0.1 ng/ml, *bars* with diagonal line upwards) or PHA-M (5 mg/ml, *bars* with spheres). HIV-1 reactivation was estimated at 12 h following reactivation, by evaluating for GFP positive cells by flow cytometry. For comparison of results across samples from multiple experiments, HIV-1 reactivation observed in vehicle control (DMSO) pretreatment was considered as 100 % and the background (no reactivating agent) as 0 %. *Error bars* represent standard deviation (N = 3), *p < 0.05

To understand whether the TFs identified in ACH-2 cells are specific to these cells or commonly shared in other HIV-1 latent cells, we validated these TFs in other HIV-1 latent cell lines, J-Lat cells that were developed by Jordan et al. [29]. First, the dose of reactivating agents that resulted in maximum response in ACH-2 cells was tested in J-Lat cells, along with PHA-M. PMA was included as a positive control. Results indicate that the cell lines FL10.6, TGA1 and TGA2 had a wide dynamic range with minor variations in their response to different reactivators (Fig. 6a). Cell line FL10.6 is highly reactive to prostratin, TNF-α and PMA reaching 60–80 % reactivation in comparison to SAHA and PHA-M (35–45 %); whereas TGA1 cells showed significant response (85–90 %) to prostratin and PMA but not to SAHA, TNF-α and PHA-M (35–60 %). The cell line TGA2 showed minimum activation with PHA-M (14–18 %) but the response to SAHA, prostratin, TNF-α and PMA were comparable to each other (~43–72 %). As previously reported these cell lines had different reactivity to different reactivators [28].

Next, we validated the specific TFs and regulatory networks in J-Lat based latency cell lines using inhibitors as described in our methods. Results indicate that pathways are similar in J-Lat and ACH-2 cells, with minor differences (Fig. 6b). JAK-STAT inhibitor, WP1066 and PKC inhibitor—Rottlerin have major effects in inhibiting reactivation of latent HIV-1 in J-Lat clones (close to background GFP level), suggesting that these signaling pathways are critical for HIV-1 LTR activity (Additional file 5: Fig. S4). ERK1/2 inhibitor U0126 and AZD6244 have a significant effect in inhibiting HIV-1 reactivation specifically in prostratin (~74–82 %), suggesting that ERK1/2 may be the major pathway involved downstream of PKC in prostratin mediated latent HIV-1 reactivation in J-Lat clones. Similarly NF-κB is the critical factor for TNF-α mediated reactivation in J-Lat cells (57–47 % inhibition with IΚK2 inhibitor V in J-Lat FL10.6 cells, 67–83 % inhibition in J-Lat TGA1 cells and 90–94 % inhibition in J-Lat TGA2 cell line). However, JNK exhibits a central role in activating HIV-1 LTR with SAHA, prostratin or TNF-α in J-Lat cells. Interestingly, it is also noted that IΚK2 inhibitor V inhibited SAHA mediated reactivation in J-Lat cells suggesting a role for NF-κB (~62–83 %). This correlates with the relatively reduced ability of SP600125 to inhibit the SAHA mediated reactivation in comparison to reactivation observed ACH-2 cells (60–70 % inhibition in J-Lat cells versus 85–95 % inhibition in ACH-2 cells). This suggests that when JNK mediated signaling is blocked in J-Lat, NF-κB related pathways could still lead to reactivation of latent HIV-1.

### Time dependent inhibition of signaling pathways and regulatory networks in J-Lat cells identify unique role for JAK- STAT early in latent virus reactivation

**Fig. 7:**
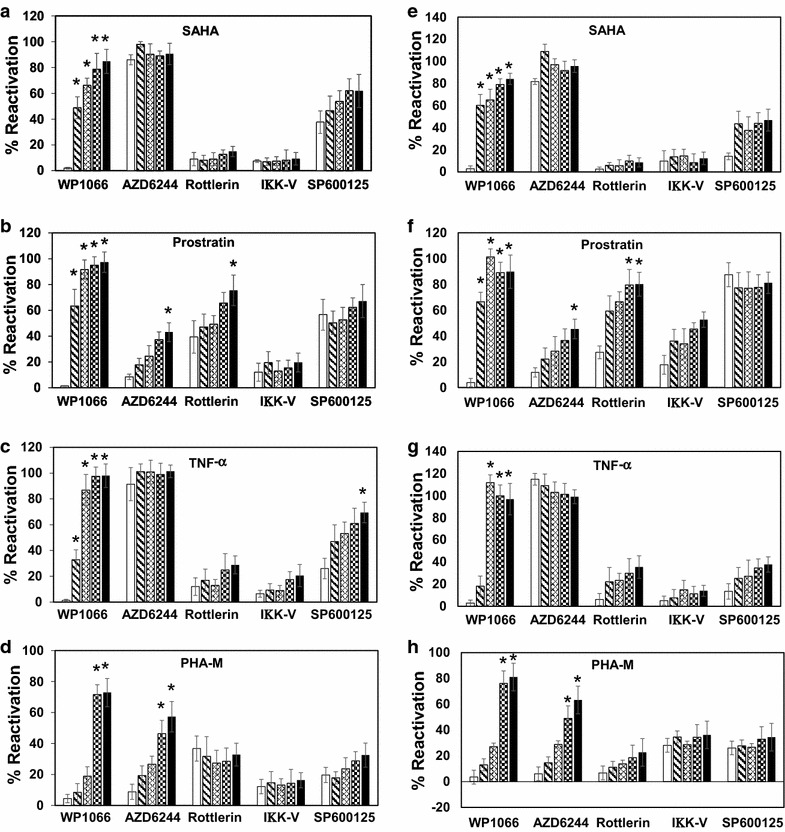
Time dependent inhibition of cellular signaling pathways in J-Lat cells. J-Lat FL10.6 (**a**–**d**) and J-Lat TGA1 (**e**–**h**) cells were activated with SAHA (1 µM), prostratin (2 µM) or TNF-α (0.1 ng/ml) or PHA-M (5 μg/ml) and specific inhibitors targeting STAT, ERK1/2, PKC, NF-κB and JNK were included at multiple time points, either 4 h prior to activation (*white bars*) or at every 2 h intervals till 8 h post activation (2 h: *bars* with diagonal downward lines; 4 h: *bars* with diagonal crossing lines; 6h: *bars* with *white* and *black* checkered pattern; 8 h: *black bars*). HIV reactivation was estimated at 18 h following reactivation, by evaluating for GFP positive cells by flow cytometry. For comparison of results across samples from multiple experiments, HIV-1 reactivation observed in vehicle control (DMSO) pretreatment was considered as 100 % and the background (no reactivating agent) as 0 %. *Error bars* represent standard deviation (N = 3)

The above results identified the requirement of specific cellular signaling pathways and regulatory network in J-Lat cells upon activation by various reactivating agents. To identify the relationship between these specific regulatory networks and their role in latent HIV reactivation, we blocked the specific regulatory network following reactivation of latent virus in a time dependent manner. J-Lat clone FL 10.6 and TGA1 were used for these experiments where the cells were stimulated with SAHA, prostratin, TNF-α or PHA-M followed by blocking of specific signaling network by using small molecule inhibitors at multiple time points of 2 h intervals. Cells treated with inhibitor 4 h prior to stimulation was included as controls, and cells stimulated with latency reactivating agents with no inhibitor were included as positive control and was normalized to 100 %. Results indicate that including the inhibitors at 4 h prior to stimulation identified specific pathways involved in latent virus reactivation as in earlier experiments (Fig. 6b, c), where we noticed >95 % inhibition with STAT inhibitor, WP1066 in both the tested J–Lat cell lines, with all the tested latency reactivating agents (Fig. 7a–h, unfilled open bars). Whereas, when WP1066 was added 2 h post stimulation, it can be noticed that the inhibition of virus reactivation was reduced to 40–50 % in both SAHA and prostratin. Including WP1066 after 4 h or later post treatment with SAHA or prostratin resulted in no effect on HIV-1 latency reversal (Fig. 7a, b, e, f). In case of TNF-α and PHA-M, the ability of WP1066 to block virus reactivation is lost by 4 and 6 h, respectively (Fig. 7c, d, g, h).

With ERK1/2 inhibitor, we observed a more gradual loss of inhibition over time (Fig. 7b, d, f, h) when prostratin and PHA-M are included to reactivated the virus. The inhibition is >95 % when AZD 6244 was included 4 h prior to activation, this inhibition gradually decreased to 40–60 % at 8 h post stimulation. JNK inhibitor, SP600125, also demonstrated consistent inhibition around 40–60 % with both the cell lines with all the tested reactivating agents but for prostratin in J-Lat TGA1cells (Fig. 7f), when included 4 h prior to stimulation. This inhibitory effect reduced progressively with time over to 40 % especially in J-Lat cell line FL10.6 (Fig. 7a–d) but did not change drastically till 8 h in J-Lat cell line TGA1. Rottlerin showed consistent inhibition (~80–90 %) over time till 8 h in both the cell lines when stimulated with SAHA, TNF-α and PHA-M, but with prostratin, the inhibitory effect of rottlerin progressively reduces to ~20 % at 8 h post stimulation (Fig. 7b, f). NF-κB inhibitor was able to consistently inhibit virus reactivation (~60–90 %) that remains stable over time in both the cells with all the tested reactivators but for in J-Lat cells TGA1 with prostratin (Fig. 7f). These results suggest that JAK-STAT has a role in early stage of reactivation, in the initial 2–4 h, and inhibiting JAK-STAT pathway after this interval does not inhibit latent virus reactivation with all the tested activators, though with delayed kinetics for TNF-α and PHA-M. And this early role of JAK-STAT pathway is essential for viral reactivation which cannot be complemented by other signal or factors. With other regulatory factors like NF-κB, PKC, JNK and ERK1/2, the activation of these factors are essential at all-time points for effective reactivation, though at later time points, the inhibition of these activated factors can complement each other, which leads to gradual loss of inhibitory activity.

### Role of signaling pathways and regulatory networks in latent virus reactivation in primary T cells

**Fig. 8:**
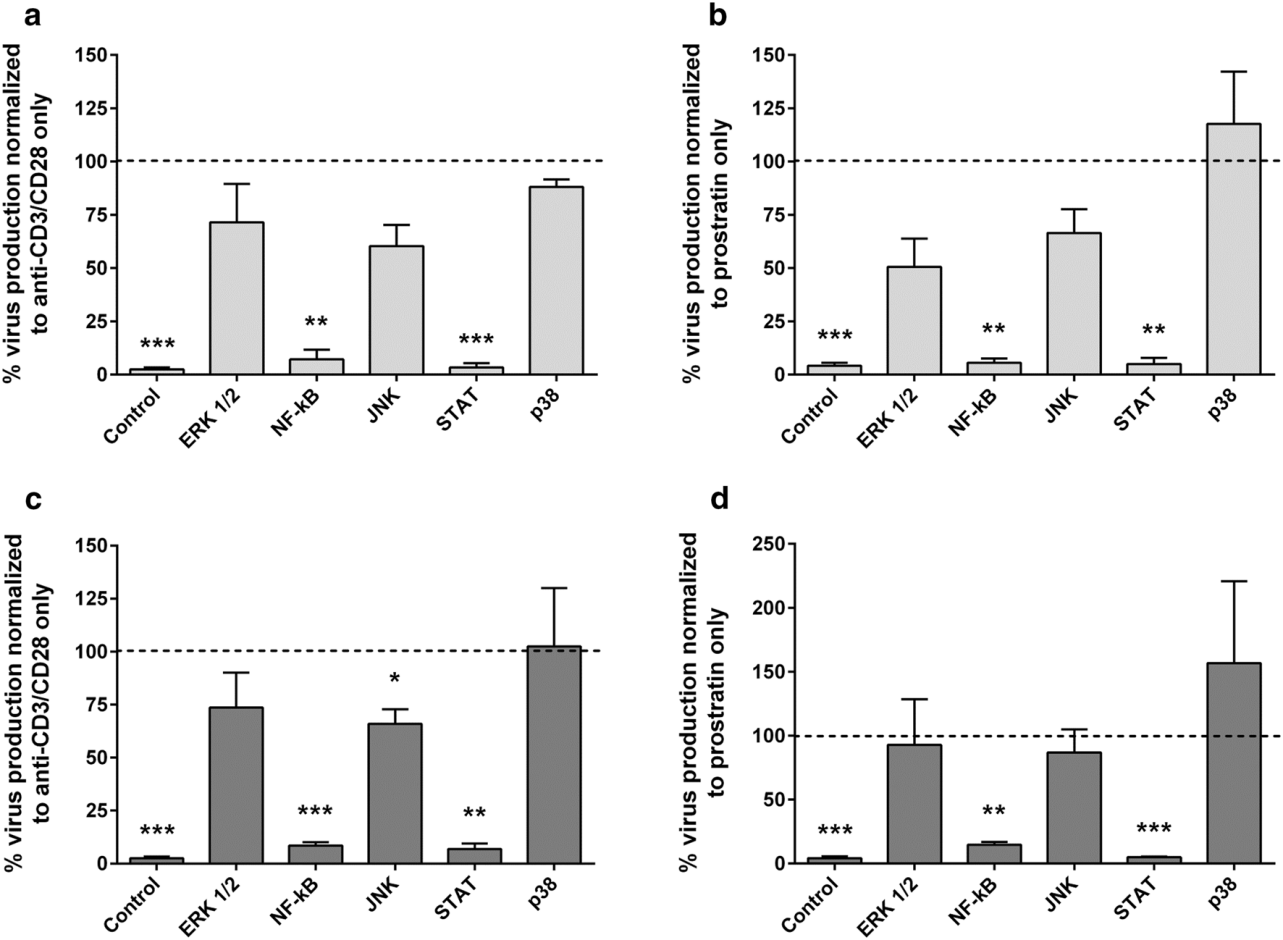
Role of specific cellular signaling pathway in reactivation of HIV-1 primary resting CD4^+^ T cells. HIV-1 latency reversal was measured by quantification of viral RNA in the culture supernatant 7 days post-stimulation with either αCD3/αCD28 (**a**, **c**) or Prostratin (**b**, **d**). Latently infected resting CD4^+^ T cells were treated with one of five pathway inhibitors: ERK1/2, NF-κB, JNK, STAT and p38, for (**a**, **b**) 4 h before stimulation or (**c**, **d**) 4 h post stimulation. Data are normalized to virus production following stimulation with (**a**, **c**) CD3/αCD28 only or (**b**, **d**) prostratin only from three independent experiments performed in duplicate are included. P values were determined using a paired t test. *Error bars* represent the standard error of the mean. *p < 0.05, **p < 0.005, ***p < 0.0005

Next we evaluated the role of identified signaling pathways and regulatory factors and the relevance of the two-phase reactivation process in primary resting CD4^+^ T cells. A total resting CD4^+^ T cell based HIV latency model was used to study the role of specific signaling pathways [30], and αCD3/αCD28 or prostratin were used to reactivate latent virus. Small molecule inhibitors targeting ERK1/2, NF-κB, JNK, STAT and p38 were included either 4 h prior to stimulation or 4 h post stimulation. The concentration of inhibitor, which induced minimal cytotoxicity (Additional file 6: Fig. S5) was included and were tested in triplicates in three independent donors (Fig. 8a–d). Results confirm a consistent critical role for JAK-STAT and NF-κB with both αCD3/αCD28 and prostratin in all the three donors, where we observed a consistent inhibition of more than 80 %. Overall, inhibition of virus reactivation by JNK inhibitor, SP600125, or ERK1/2 inhibitor AZD6244, or P38 inhibitor, SB203580 were not statistically significant in resting T cells; however, JNK inhibitor, SP600125, inhibited virus reactivation by 40–55 % in two of the three donors tested with both αCD3/αCD28 and prostratin. Similarly ERK1/2 inhibitor, AZD6244 had inconsistent effect where ~55–60 % inhibition was observed in one donor with both the tested reactivators. Inhibiting specific pathways either 4 h prior to or after addition of reactivating agents did not consistently alter the inhibition of virus reactivation. WP1066 and IΚK2 inhibitor V consistently inhibited virus reactivation >80 % in all the tested donors (Fig. 8a–d), suggesting that JAK-STAT, NF-κB are required also at 4 h following stimulation with prostratin or αCD3/αCD28 for latent virus reactivation in primary T cells.

## Discussion

Inability to consistently reactivate the latent viral reservoir is presently the main impediment in the “shock and kill” strategy to achieve HIV-1 cure. Numerous therapeutic agents and methods have been proposed to reactivate the latent virus but the results vary widely between individual patients, suggesting that multiple factors are involved in determining the reactivation of latent HIV-1. This variability in outcomes is true for in vitro primary CD4^+^ T cell based models as well as established T cell lines, where different activators are effective to reactivate latent virus to variable extent. Hence there is an urgent need to develop innovative approaches to predict and identify strategies that could result in more effective reactivation and eventual viral elimination. Here in this study, using ACH-2, a T cell line (that has a single latent copy of replication competent provirus), we describe the application of a robust Systems Biology approach to predict factors that are potentially involved in latent HIV-1 reactivation. Changes in transcripts of NT5C3 gene in ACH-2 cells suggest that onset of HIV latency in these cell lines is associated with silencing of the host gene where the virus integrated and can be induced upon virus reactivation, suggesting methods by which a retroviral genetic element can regulate host cellular expression. Additionally these changes indicate that HIV latency and reactivation in ACH-2 cells is associated with local chromosomal changes that are specific to the region of virus integration, which could be due changes in the nucleosomal assembly and/or DNA methylation.

Using Systems Biology method, we integrate several genome wide transcriptome datasets to reconstruct a dynamic signaling and regulatory network for each reactivating agent. This helped to specifically identify the critical factors for individual treatments along with their time of activation and the specific set of genes regulated by these critical factors. We validated our approach using specific inhibitors to confirm the cellular factors that are critical for latent HIV-1 reactivation. The latent HIV-1 virus in ACH-2 cells is integrated in chromosome 7 and has a point mutation in TAR- Tat axis C37T. It has been previously identified that the TAR mutation renders the virus less responsive to Tat and suggests that Tat-TAR axis may have a role in HIV-1 latency in patients [48]. The mutation in TAR region helps us to understand the cellular pathways involved in HIV-1 latency reversal in the early phase in the absence of viral protein Tat. Additional reports also suggest a role for DNA methylation in LTR region of HIV-1 virus identified in ACH-2 as the major contributing factor for latency and reactivation from latency [47]. Therefore, results from ACH-2 cells were further confirmed in J-Lat cell clones and primary T cell HIV latency model that do not contain any mutations and similar results were observed, thus confirming the validity of the cell lines as latent cell model. SDREM reconstructs accurate models of the cross talk between signaling pathways and transcriptional regulatory networks within cells, which are essential to understand complex host cellular response programs [49]. This computational method combines condition-specific time series expression data with general protein interaction data to reconstruct dynamic and causal networks [50, 51]. Similar analyses were performed in H1N1 influenza infection to identify strain specific targets of Influenza infection [39].

Analyses of time kinetic transcriptome data corresponding to the initial period of reactivation extending up to 6 h in ACH-2 cells post treatment with SAHA, clearly identified Jun as the key regulatory factor that is responsible for the increase in cellular transcripts observed in the initial 2 h. SDREM also predicts NFATC1-4, CEBPE, TBP and ATF2 as potential factors modulating cellular transcript levels. These factors are known to bind to HIV-1 LTR and have a role in HIV-1 transcription. However, interpretation of predicted factors associated with a decrease in cellular transcript level is complex, since it can mean that either association or dissociation of these predicted TFs may lead to suppression of cellular transcripts). Some of the other top 30 factors regulated by SAHA include—STATs, TP53, RB1, NF-κB related factors, BRCA1, NR3C1, CTNNB1, SMADs and Myc (Table 2). Though these factors are part of the predicted regulatory network, validating the role of these factors is difficult, as targeting specific cellular factors can result in cells using alternative compensatory networks, and multiple TFs can be regulated by a single upstream regulatory pathway, hence the recommended approach includes targeting specific signaling or regulatory pathways rather than TFs. The SDREM prediction for SAHA highly correlates with the results in validation experiments, where Jun is identified as the critical cellular factor and validation experiments using JNK inhibitor SP600125 inhibits SAHA induced reactivation of HIV-1 in ACH-2 cells, and J-Lat cell lines supporting the validity of these analyses.

Multiple factors, including ETS1, CEBP, STATs, RELA and GATA 2/3, which are known to bind to HIV-1 LTR, are predicted by SDREM as active in ACH-2 cells within the initial 4 h following prostratin treatment. The predicted regulators that are unique for prostratin include MAPK1, SMADs2/4 and others. This prediction correlates well with the results obtained in validation experiments, where we observed specific inhibition of prostratin induced HIV-1 reactivation in the presence of ERK1/2, NF-κB pathway and JNK inhibitors. The SDREM prediction is also accurate for TNF-α treatment, which identified NF-κB related factors, STATs, CEBP, ETS1 and GATA3, and the validation results correlated with the inhibitory effect observed with IΚK-2 inhibitor V and SP600125. Additionally, it can be noted that STATs are common in the network for all the three treatments and correlate with the inhibitory effect of WP1066, though STAT may also have a role in regulation of basal HIV-1 LTR transcription, as we observed a decrease in p24 positive cells with WP1066 in the absence of latency reactivating agents. Similar results describing a central role for STAT were also reported in acute HIV-1 infection and in reactivation of latent HIV infection by using STAT inhibitors Ruxolitinib and Tofacitinib [52]. Our time kinetics data in J-Lat cell lines further refines the role of STAT and restricts the requirement of JAK-STAT pathway to the initial immediate early stage of latent HIV reactivation. Other factors that are predicted in common for all three treatments include BRCA1, CEBPB, JUN, MYC, NR3C1, RELA, SMAD3, TP53 and UBC. Previous results suggest that Myc may have a role proviral latency by recruiting Histone Deacetylase 1 to the HIV-1 promoter [19], and we also found that c-Myc levels are reduced in the three treatments tested in ACH-2 cells, which may have a role in HIV-1 reactivation. The role of other factors identified as common or unique needs to be evaluated in patients.

The minor differences in degree of response and the cellular pathway involved are expected in different cell types. Previously it has been demonstrated that the latent HIV-1 virus in the human monocytic cell line U1 can be induced by cytokines, including TNF-α, which is sensitive to U0126 and has been suggested to involve cooperative interaction of AP-1 and NF-κB at the HIV-1 LTR [53]. Our results though suggests that AP-1 and NF-κB have a critical role in TNF-α induced reactivation of latent virus in ACH-2 and J-Lat cells, but this does not require MAPK as U0126 was unable to inhibit the TNF-α mediated reactivation of latent HIV-1. This could be explained as the availability of alternate signaling pathways in different cell types, which might compensate for missing factors. Also, in the primary T cell model developed by Bosque et al. [54], it was shown that Cyclosporin A and p38 MAPK inhibitor inhibited αCD3/αCD28 or PHA-M mediated reactivation of latent HIV-1, but in ACH-2 cells, NFAT and p38-MAPK did not seem to have a role in latent HIV-1 reactivation, though NFAT seems to have a minor role in PHA-M induced latent virus reactivation in J-Lat clones. In our primary resting T cell model, we see a major role for JAK-STAT and NFκB in reversal of latency mediated by αCD3/αCD28 or prostratin, with variable role for JNK and ERK1/2 which were found to have a minor role in some donors but not all and the inhibition was not statistically significant. SB203580, p38 MAPK inhibitor, did not show any inhibition in our primary T cell model, suggesting no role for p38 in latent virus reactivation. Similar variations were observed when reactivity of different in vitro primary T cell models and cell lines were compared [22]. SAHA was not tested in our primary cell model, as it is ineffective and does not reactivate latent HIV-1 as has been observed in in vitro studies using resting CD4^+^ T cells isolated from aviremic infected individuals [55–57]. Though ACH-2 cells have good dynamic range in their response to LRAs (Latency Reversing Agents), it can also be noted that the background of non-latent virus containing cells in ACH-2 population is around ~13–20 %. This background could potentially undermine the magnitude of fold change observed in cellular transcripts induced by LRAs at the population level. A twofold change in transcripts in the 75–80 % of cells will be observed as 1.5–1.6 fold change at the population. However, SDREM considers the changes observed in global transcripts over time that are independent of the magnitude of fold change to predict the upstream regulating factors, hence the reduced fold changes in transcripts does not affect the analyses.

**Fig. 9:**
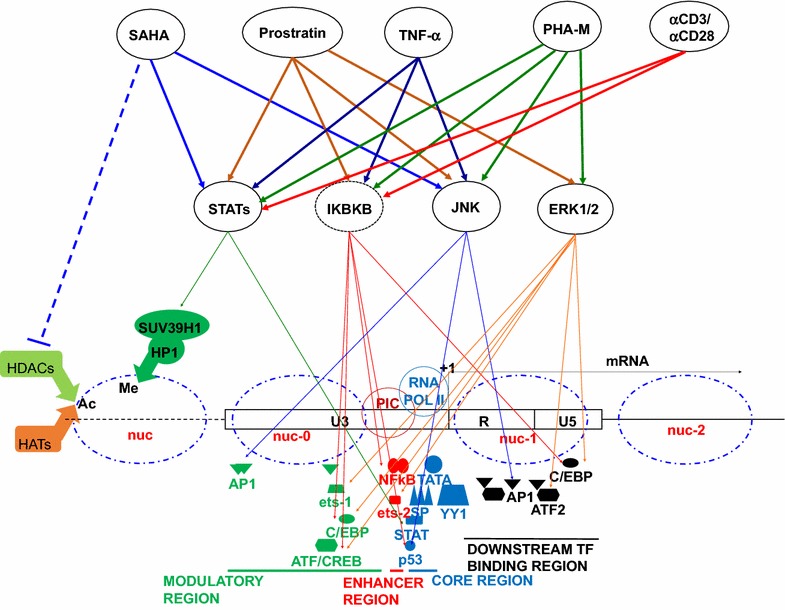
Model depicting the cellular signaling pathways involved with tested Latency Reactivating Agents. SAHA, prostratin, TNF-α, PHA-M were tested in ACH-2 cells and J-Lat cell clones. αCD3/αCD28 was tested only in primary T cell model. Prostratin when tested in primary T cell model, suggested a role only for STAT and NFΚB. IΚBKB degradation leads to activation of downstream transcription regulators. Only few TFs that have binding sites on LTR corresponding to core, enhancer, modulatory and downstream regions are represented, the list of possible signaling molecules that can directly or indirectly regulate HIV-1 latency status is included in Table 2. *Ac* acethylation, *Me* methylation, *HDAC* histone deacetylase, *HAT* histone acetyl transferase, *SUV39H1* histone-lysine *N*-methyltransferase, *HP1* heterochromatin protein 1, *nuc* nucleosome, *PIC* preinitiation complex, *RNA POLII* RNA polymerase II)

Temporal inhibition of signaling pathways and regulatory network post activation in J-Lat cells identify a biphasic mechanism where JAK-STAT pathway has an essential role in the initial stage and the second phase does not require JAK-STAT, whereas, the other signaling pathways have a role. With the current results, it is not possible to identify if JAK-STAT acts independently in the initial phase or in concert with other regulatory molecules. Interaction of STAT with NF-κB has been well studied in cancer biology [58], however their role in HIV-1 transcriptional regulation and the mechanisms are unclear. Previous reports suggest that disassembly of nucleosome, degradation of myc are part of the early essential events during reactivation of latent virus, hence an understanding of the relationship between these events and JAK-STAT may help to dissect the details of immediate early events of reactivation. Also HIV-1 LTR has putative sites for STAT binding and has been identified as essential factor for efficient LTR transactivation [59], mutation of these sites may help us understand if STAT has a role in early transcription initiation along with other known transcription factors regulating HIV-1 LTR activity. In summary as depicted in a simplistic schematic model (Fig. 9), it is possible to predict that JAK-STAT either independently or in concert with other regulatory factors helps in the immediate early phase of reactivation where JAK-STAT has an essential role, which is followed by the next phase which, is JAK-STAT independent phase with less fidelity in the choice of regulatory factors driving LTR transcription. TFs that have binding sites in core, enhancer, modulatory or downstream TF binding region can alter the activity of stalled RNA polymerase at HIV-1 LTR promoter or aid in overcoming the reversal of inhibitory TF effects. Transcriptional interference has also been reported as one of the mechanisms contributing to HIV-1 latency, hence these regulatory TF activation can help to overcome the transcriptional interference and associated reversal of latency.

Surprisingly, this temporal requirement of JAK-STAT was not observed at 4 h with prostratin or αCD3/αCD28 in primary T cell model suggesting that the absence could be due to delayed kinetics of the JAK-STAT dependent phase of virus reactivation. This is true in J-Lat cells when they were reactivated with TNF-α and PHA-M, where JAK-STAT dependent phase extended till 6 h post activation. Furthermore, within an individual, different latent reservoirs might respond differently based on the availability of cellular signaling pathways and other factors influencing the signal transduction. Given the short duration time kinetics data required and the small amount of cells required to obtain transcriptome data, SDREM has potential to be adapted to characterize cellular signaling pathways—to identify defects in pathways of HIV-1 subjects, and to identify alternate pathways that can bypass the defects to reactivate the HIV-1 transcription with minimal adverse effects. Also, the SDREM approach will help in assay development—to compare functional signaling pathways in cell models that can closely simulate conditions present in cells obtained from latent reservoir of HIV-1 patients.

## Conclusion

Our results support the wide variations observed in outcomes both in patients and CD4^+^ T cell models, suggesting that cells derived from HIV-1 infected patients may respond differently to various activators and that a single strategy may not be optimal for all individuals, though JAK-STAT and NF-κB seems to be the common regulatory factors essential for latent HIV reactivation. JAK-STAT has an essential role in immediate early phase of HIV-1 latency reversal, where activation of STAT is critical for reversal of HIV-1 latency, though the duration of this immediate early phase varies between latent HIV-1 cell models tested and the LRAs used. Other cellular signaling pathways including NFκB, NFAT, JNK, and ERK1/2 are also involved in latent HIV-1 reversal and could play a complement role with each other that is essential during reactivation. SDREM based prediction of cellular signaling pathways will help to optimize the management of HIV-1 patients to achieve the best outcome and cure.

## Additional files

10.1186/s12977-015-0211-3 Live cells were gated based on Forward Scatter and Side Scatter and the expression of CD2, CD3 and CD4 in ACH-2 cells was analyzed by flow cytometry using directly conjugated specific antibodies and isotype controls.
10.1186/s12977-015-0211-3 Fold change in expression of NT5C3 transcripts at 18 hours in ACH-2 cells following treatment with SAHA, prostratin or TNF-α.
10.1186/s12977-015-0211-3 Reversal of HIV-1 latency in ACH-2 cells is associated with induction of NT5C3 gene expression. (**A**) Schematic representation of organization of genes on Chromosome 7 between nucleotides 32916815 to 33606068. (**B**) ACH-2 cells were treated with prostratin, SAHA or TNF-α and the average fold change in expression of NT5C3 transcripts over time relative to time 0 as evaluated by illumina HT-12 V4 array bead chips was included (N = 2). (**C**) Western blot analysis of NT5C3 in ACH-2 cells or A3.01 cells post-treatment with SAHA, prostratin or TNF-α. At indicated time points, the cells were washed and lysed. Equal amounts of cell lysate were analyzed by immunoblotting them with anti-NT5C3 or anti-tubulin antibodies. NT5C3 antibody also detects NT5C3L protein.
10.1186/s12977-015-0211-3 ACH-2 cells were pretreated with multiple doses of (**A**) Rottlerin and (**B**) WP1066 or vehicle control (DMSO), and four hours later activated with SAHA (1 µM, white bars), prostratin (1 µM, grey bars) or TNF-α (0.1 ng/ml, bars with diagonal line upwards). HIV-1 reactivation was estimated at 12 h following treatment, by intracellular p24 Gag staining by flow cytometry. For comparison of results across samples from multiple experiments, HIV-1 reactivation observed in vehicle control pretreatment was considered as 100 % and the background (no reactivating agent) as 0 %. Error bars represent standard deviation (N = 3).
10.1186/s12977-015-0211-3 J-Lat cells FL10.6, TGA1 and TGA2 were treated with multiple doses of (**A**) Rottlerin and (**B**) WP1066 or vehicle control (DMSO), and 16 h post-treatment, the live cells were evaluated by Trypan blue staining. The percentage of viable cells was calculated by subtracting the dead cells from total cells divided by total cell count. Viability of cells in the vehicle control was considered as 100 % for comparison across three independent experiments. Error bars represent standard deviation (N = 3).
10.1186/s12977-015-0211-3 Primary resting CD4^+^ T cells were treated with multiple concentrations of small molecules inhibiting specific cellular signaling pathway. Cell viability was evaluated after 3 days by flow cytometry and trypan blue staining. Results from two independent donors tested in duplicates that are normalized to DMSO only control are included.

## Abbreviations

HIV-1: human immunodeficiency virus type 1
LRAs: latency reversing agents
cART: combined antiretroviral therapy
MS-RNA: multiply spliced RNA
SDREM: signaling and dynamic regulatory events miner
TFs: transcription factors
SAHA: suberoylanilide hydroxamic acid
PHA-M: phytohemagglutanin-M
TNF-α: tumor necrosis factor-α
CsA: cyclosporin A
PKC: protein kinase C
PMA: phorbol 12-myristate 13-acetate
RPLPO: ribosomal large protein
PPI: protein–protein interaction
Ac: acethylation
Me: methylation
HDAC: histone deacetylase
HAT: histone acetyl transferase
SUV39H1: histone-lysine *N*-methyltransferase
HP1: heterochromatin protein 1
Nuc: nucleosome
PIC: preinitiation complex
RNA POLII: RNA polymerase II
