# Supplementary figures and images for: Temporal transcriptional response to latency reversing agents identifies specific factors regulating HIV-1 viral transcriptional switch
Source: Retrovirology. 2015 Oct 6;12:85. doi: 10.1186/s12977-015-0211-3 (PMC4594640; doi:10.1186/s12977-015-0211-3)

## Slide 1
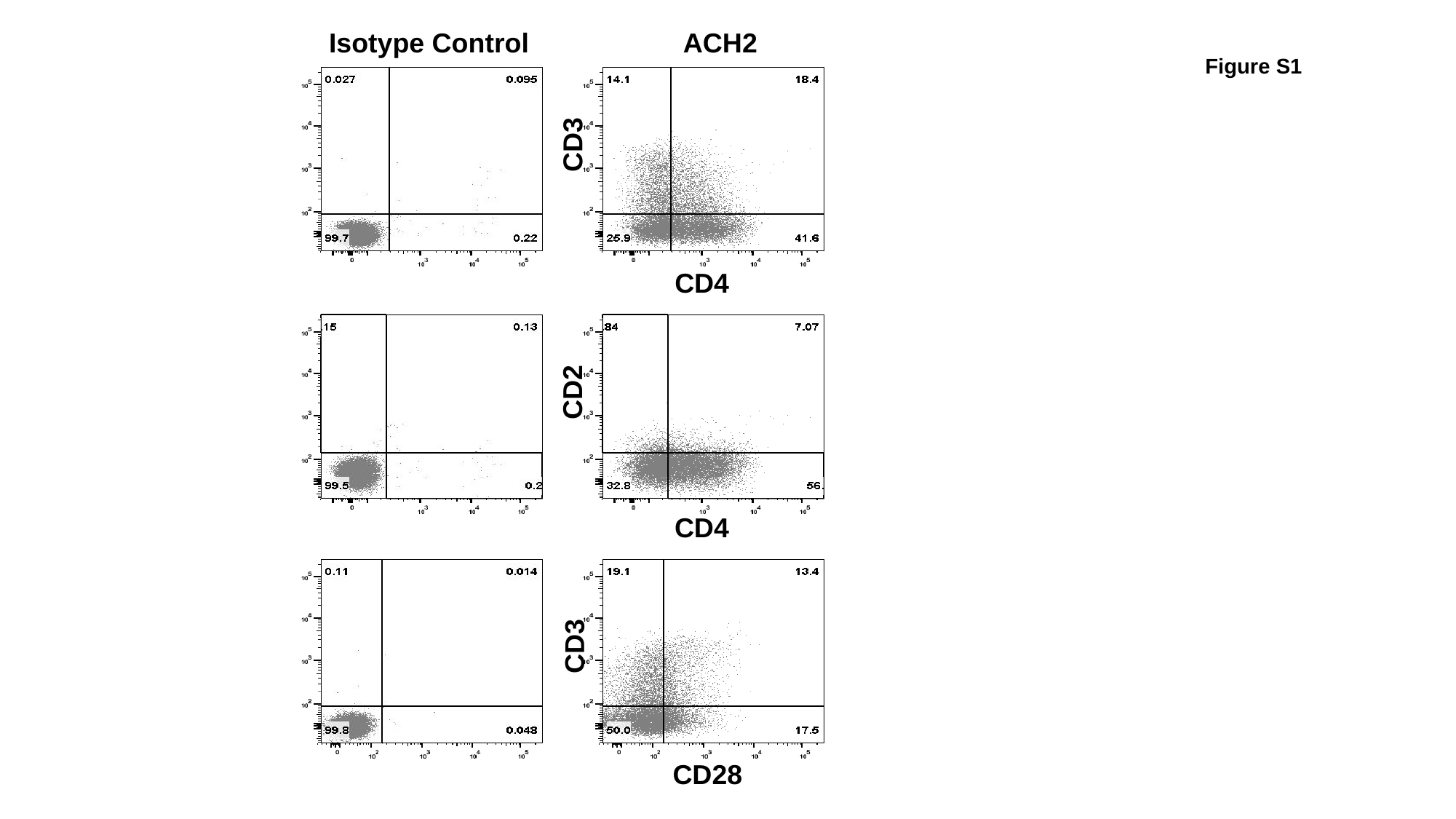

Isotype Control
ACH2
CD3
CD4
CD2
CD4
CD3
CD28
Figure S1

Supplement: Supplementary file 1 — 10.1186/s12977-015-0211-3 Live cells were gated based on Forward Scatter and Side Scatter and the expression of CD2, CD3 and CD4 in ACH-2 cells was analyzed by flow cytometry using directly conjugated specific antibodies and isotype controls. [file 12977_2015_211_MOESM1_ESM.pptx]
