# Supplementary material for: Temporal transcriptional response to latency reversing agents identifies specific factors regulating HIV-1 viral transcriptional switch
Source: Retrovirology. 2015 Oct 6;12:85. doi: 10.1186/s12977-015-0211-3 (PMC4594640; doi:10.1186/s12977-015-0211-3)
Supplement: Supplementary file 2 — 10.1186/s12977-015-0211-3 Fold change in expression of NT5C3 transcripts at 18 hours in ACH-2 cells following treatment with SAHA, prostratin or TNF-α. [file 12977_2015_211_MOESM2_ESM.docx]

**Table. S1:** Fold change in expression of NT5C3 transcripts at 18 hours in ACH-2 cells following treatment with SAHA, prostratin or TNF-α.

| **Gene** | **Latency reversing agent** | **Fold change** | **Diff-score** |
| --- | --- | --- | --- |
| **NT5C3** | Prostratin | 12.6 | 217 |
|  | SAHA | 18.5 | 354 |
|  | TNF-α | 7.4 | 339 |
| **BBS9** | Prostratin | 1.0 | 0.0 |
|  | SAHA | 1.1 | 4.8 |
|  | TNF-α | 1.0 | 0.0 |
| **FKBP9** | Prostratin | 1.0 | -0.1 |
|  | SAHA | 1.0 | 0.0 |
|  | TNF-α | 1.0 | 0.0 |
| **RP9** | Prostratin | 1.1 | 0.0 |
|  | SAHA | 1.4 | 0.0 |
|  | TNF-α | 0.9 | 0.0 |
| **RP9P** | Prostratin | 1.0 | 0.0 |
|  | SAHA | 1.0 | 0.0 |
|  | TNF-α | 1.0 | 0.0 |

Comparison of cellular transcripts of genes present on chromosome 7 near the site of proviral integration in ACH-2 cells, at 18 hours in comparison to time 0 (before addition of latency reversing agents).
