# Supplementary material for: Temporal transcriptional response to latency reversing agents identifies specific factors regulating HIV-1 viral transcriptional switch
Source: Retrovirology. 2015 Oct 6;12:85. doi: 10.1186/s12977-015-0211-3 (PMC4594640; doi:10.1186/s12977-015-0211-3)
Supplement: Supplementary file 3 — 10.1186/s12977-015-0211-3 Reversal of HIV-1 latency in ACH-2 cells is associated with induction of NT5C3 gene expression. (A) Schematic representation of organization of genes on Chromosome 7 between nucleotides 32916815 to 33606068. (B) ACH-2 cells were treated with prostratin, SAHA or TNF-α and the average fold change in expression of NT5C3 transcripts over time relative to time 0 as evaluated by illumina HT-12 V4 array bead chips was included (N = 2). (C) Western blot analysis of NT5C3 in ACH-2 cells or A3.01 cells post-treatment with SAHA, prostratin or TNF-α. At indicated time points, the cells were washed and lysed. Equal amounts of cell lysate were analyzed by immunoblotting them with anti-NT5C3 or anti-tubulin antibodies. NT5C3 antibody also detects NT5C3L protein. [file 12977_2015_211_MOESM3_ESM.pptx]

## Slide 1
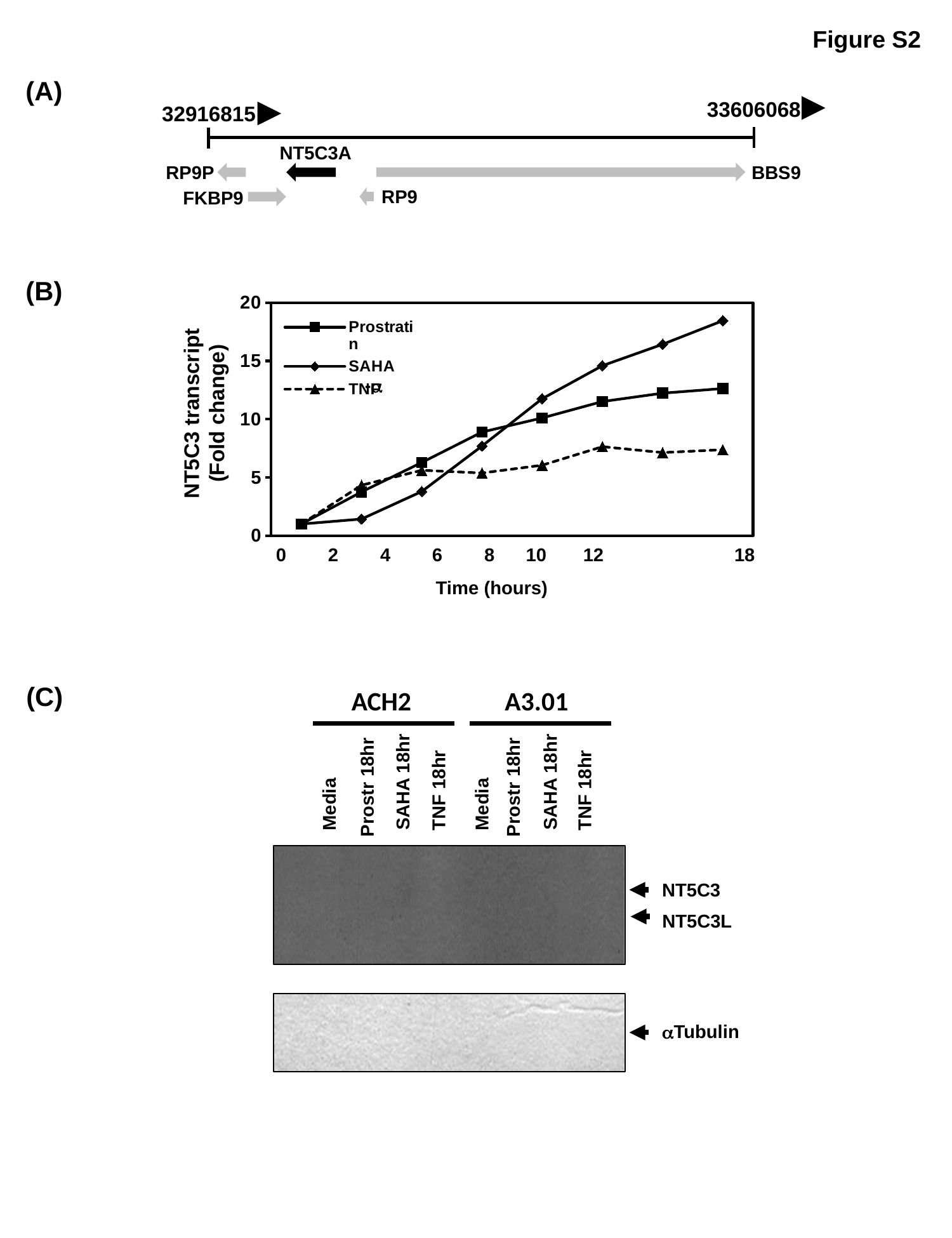

Figure S2
(A)
33606068
32916815
NT5C3A
RP9P
BBS9
RP9
FKBP9
### Chart
| Category | Prostratin | SAHA | TNF |
|---|---|---|---|
| 0 | 1.0 | 1.0 | 1.0 |
| 2 | 3.755948 | 1.426683 | 4.329658 |
| 4 | 6.279247 | 3.790688 | 5.612669 |
| 6 | 8.890663 | 7.691461 | 5.378158 |
| 8 | 10.10572 | 11.75409 | 6.047976999999999 |
| 10 | 11.51357 | 14.59053 | 7.638098 |
| 12 | 12.23194 | 16.42705 | 7.132018999999999 |
| 18 | 12.62934 | 18.45503 | 7.376403 |0 2 4 6 8 10 12 18
NT5C3 transcript
(Fold change)
Time (hours)
(B)
-a
(C)
ACH2
A3.01
SAHA 18hr
SAHA 18hr
Prostr 18hr
Prostr 18hr
TNF 18hr
TNF 18hr
Media
Media
NT5C3
NT5C3L
aTubulin
