# Supplementary material for: Temporal transcriptional response to latency reversing agents identifies specific factors regulating HIV-1 viral transcriptional switch
Source: Retrovirology. 2015 Oct 6;12:85. doi: 10.1186/s12977-015-0211-3 (PMC4594640; doi:10.1186/s12977-015-0211-3)
Supplement: Supplementary file 4 — 10.1186/s12977-015-0211-3 ACH-2 cells were pretreated with multiple doses of (A) Rottlerin and (B) WP1066 or vehicle control (DMSO), and four hours later activated with SAHA (1 µM, white bars), prostratin (1 µM, grey bars) or TNF-α (0.1 ng/ml, bars with diagonal line upwards). HIV-1 reactivation was estimated at 12 h following treatment, by intracellular p24 Gag staining by flow cytometry. For comparison of results across samples from multiple experiments, HIV-1 reactivation observed in vehicle control pretreatment was considered as 100 % and the background (no reactivating agent) as 0 %. Error bars represent standard deviation (N = 3). [file 12977_2015_211_MOESM4_ESM.pptx]

## Slide 1
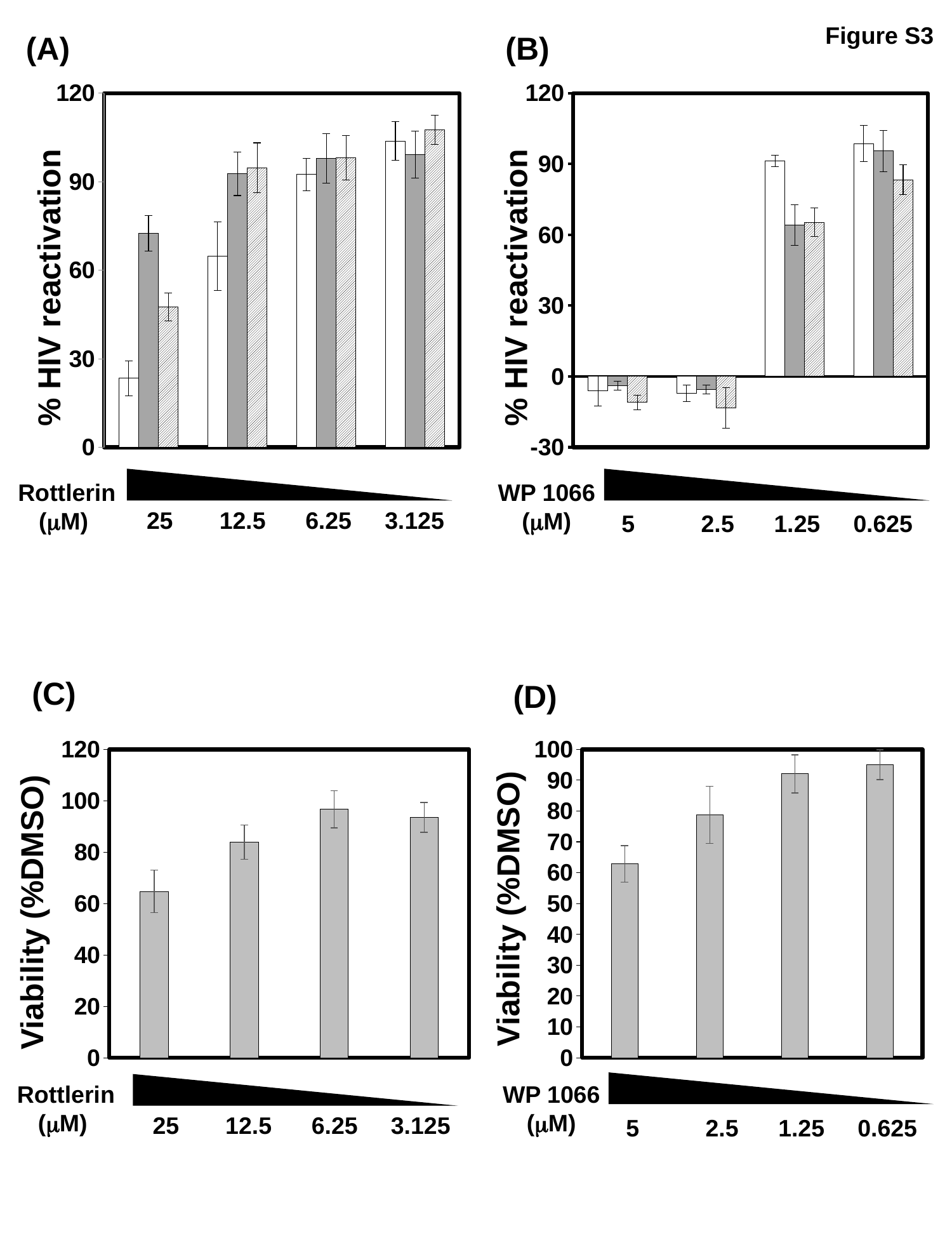

Figure S3
(A)
(B)
### Chart
| Category | S | P | T |
|---|---|---|---|
| Rott 50 | 23.35216572504708 | 72.47386759581882 | 47.59725400457665 |
| Rott 25 | 64.7834274952919 | 92.6829268292683 | 94.73684210526315 |
| Rott 12.5 | 92.46704331450094 | 97.90940766550523 | 98.077803203661 |
| Rott 6.25 | 103.747645951035 | 99.1869918699187 | 107.597254004577 |
### Chart
| Category | S | P | T |
|---|---|---|---|
| WP5 | -6.026365348399247 | -3.9140534262485485 | -11.006864988558354 |
| Wp2.5 | -7.193973634651601 | -5.563298490127759 | -13.386727688787186 |
| WP1.25 | 91.33709981167608 | 64.11149825783971 | 65.21739130434783 |
| WP0.625 | 98.68173258003766 | 95.47038327526131 | 83.29519450800915 |% HIV reactivation
% HIV reactivation
Rottlerin
(mM)
WP 1066
(mM)
25 12.5 6.25 3.125
5 2.5 1.25 0.625
(C)
(D)
### Chart
| Category | |
|---|---|
| Rottlerin 25 | 64.73042 |
| Rottlerin 12.5 | 83.873902 |
| Rottlerin 6.25 | 96.652318 |
| Rottlerin 3.125 | 93.526298 |
### Chart
| Category | |
|---|---|
| WP1066 5 | 62.88322 |
| WP1066 2.5 | 78.757198 |
| WP1066 1.25 | 92.048028 |
| WP1066 0.625 | 95.026328 |Viability (%DMSO)
Viability (%DMSO)
Rottlerin
(mM)
WP 1066
(mM)
25 12.5 6.25 3.125
5 2.5 1.25 0.625
