# Supplementary material for: Temporal transcriptional response to latency reversing agents identifies specific factors regulating HIV-1 viral transcriptional switch
Source: Retrovirology. 2015 Oct 6;12:85. doi: 10.1186/s12977-015-0211-3 (PMC4594640; doi:10.1186/s12977-015-0211-3)
Supplement: Supplementary file 5 — 10.1186/s12977-015-0211-3 J-Lat cells FL10.6, TGA1 and TGA2 were treated with multiple doses of (A) Rottlerin and (B) WP1066 or vehicle control (DMSO), and 16 h post-treatment, the live cells were evaluated by Trypan blue staining. The percentage of viable cells was calculated by subtracting the dead cells from total cells divided by total cell count. Viability of cells in the vehicle control was considered as 100 % for comparison across three independent experiments. Error bars represent standard deviation (N = 3). [file 12977_2015_211_MOESM5_ESM.pptx]

## Slide 1
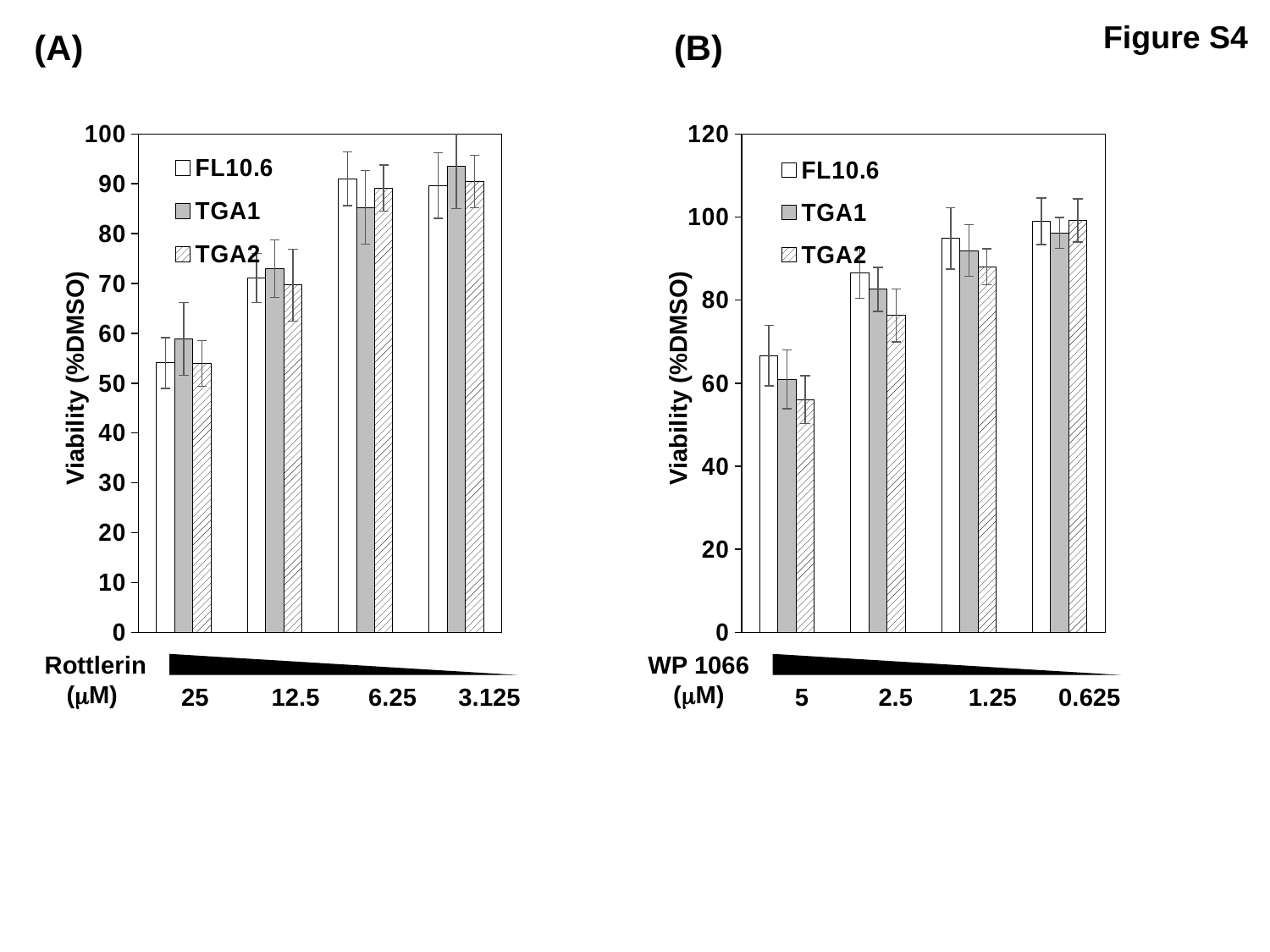

Figure S4
(A)
(B)
### Chart
| Category | FL10.6 | TGA1 | TGA2 |
|---|---|---|---|
| Rottlerin 50 | 54.0740722 | 58.8633187 | 53.9661187 |
| Rottlerin 25 | 71.087146 | 72.98204600000001 | 69.68364600000001 |
| Rottlerin 12.5 | 90.99466799999999 | 85.26466799999999 | 89.15740799999999 |
| Rottlerin 6.25 | 89.649548 | 93.445782 | 90.46108199999999 |
### Chart
| Category | FL10.6 | TGA1 | TGA2 |
|---|---|---|---|
| WP1066 5 | 66.6655887 | 60.9257887 | 56.0512267 |
| WP1066 2.5 | 86.5204958 | 82.62337231000001 | 76.32497231 |
| WP1066 1.25 | 94.834267 | 91.93686699999999 | 88.04412699999999 |
| WP1066 0.625 | 98.959128 | 96.195728 | 99.180428 | Viability (%DMSO)
 Viability (%DMSO)
Rottlerin
(mM)
WP 1066
(mM)
25 12.5 6.25 3.125
5 2.5 1.25 0.625
