# Supplementary material for: Temporal transcriptional response to latency reversing agents identifies specific factors regulating HIV-1 viral transcriptional switch
Source: Retrovirology. 2015 Oct 6;12:85. doi: 10.1186/s12977-015-0211-3 (PMC4594640; doi:10.1186/s12977-015-0211-3)
Supplement: Supplementary file 6 — 10.1186/s12977-015-0211-3 Primary resting CD4+ T cells were treated with multiple concentrations of small molecules inhibiting specific cellular signaling pathway. Cell viability was evaluated after 3 days by flow cytometry and trypan blue staining. Results from two independent donors tested in duplicates that are normalized to DMSO only control are included. [file 12977_2015_211_MOESM6_ESM.pptx]

## Slide 1
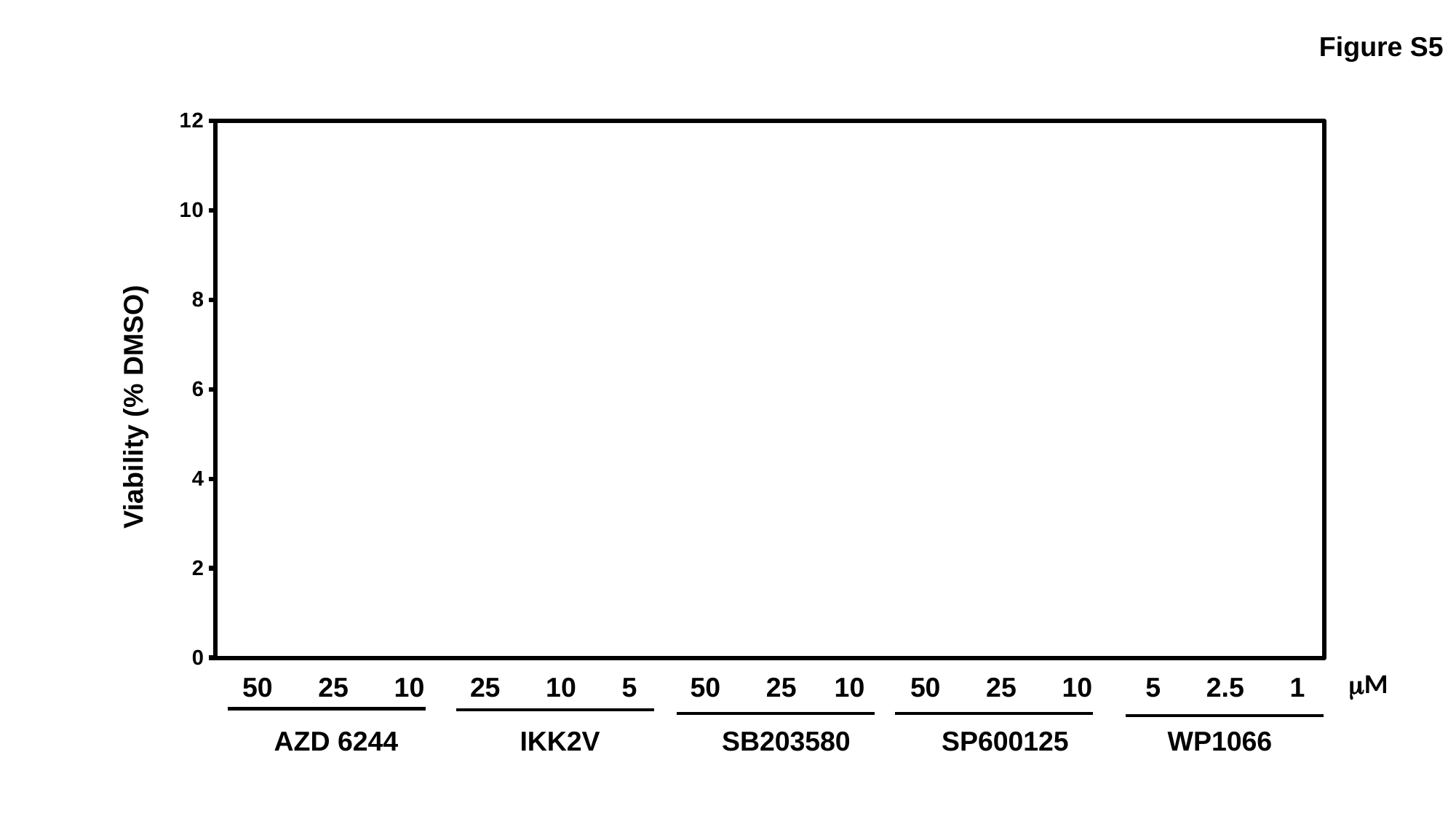

Figure S5
### Chart
| Category | |
|---|---|
| AZD6422 | 106.9363447542128 |
| AZD6422 | 103.7253353120935 |
| AZD6422 | 105.4767950077949 |
| IKKV | 113.8448802205905 |
| IKKV | 121.9210554841025 |
| IKKV | 112.3853304741727 |
| SB203580 | 95.55185673215372 |
| SB203580 | 92.53545392289024 |
| SB203580 | 103.43342536281 |
| SP600125 | 115.0125200177248 |
| SP600125 | 125.0347616097939 |
| SP600125 | 134.3758799868679 |
| WP | 101.5846623506807 |
| WP | 97.78983300999441 |
| WP | 98.56825954141728 |Viability (% DMSO)
mM
50 25 10 25 10 5 50 25 10 50 25 10 5 2.5 1
AZD 6244 IKK2V SB203580 SP600125 WP1066
